# Exploiting branched-chain amino acid metabolism and NOTCH3 expression to predict and target colorectal cancer progression

**DOI:** 10.3389/fimmu.2024.1430352

**Published:** 2024-09-02

**Authors:** Kuan Shen, Chuming Zhu, Jianjun Wu, Jiang Yan, Pengyu Li, Shuqing Cao, Xinyi Zhou, Guozhong Yao

**Affiliations:** ^1^ Department of General Surgery, Liyang People’s Hospital, Liyang Branch Hospital of Jiangsu Province Hospital, Liyang, China; ^2^ Department of General Surgery, the First Affiliated Hospital of Nanjing Medical University, Nanjing, China

**Keywords:** branched-chain amino acids, colorectal cancer, single-cell sequencing, tumor microenvironment, NOTCH3

## Abstract

**Background:**

The interplay between colon adenocarcinoma (COAD) and branched-chain amino acid (BCAA) metabolism is not fully understood, presenting a crucial area for investigation.

**Methods:**

We developed a prognostic model based on BCAA metabolism using the least absolute shrinkage and selection operator (LASSO) regression algorithm. We employed qRT-PCR and Western blot analyses to examine NOTCH3 expression in COAD tissues versus adjacent non-cancerous tissues and various cell lines. We also investigated the impact of NOTCH3 on COAD cell proliferation, invasion, and migration through *in vitro* and *in vivo* experiments.

**Results:**

Our BCAA metabolism-related signature (BRS) distinguished between different immune features, tumor mutation burdens, responses to immunotherapy, and drug sensitivity among COAD patients. NOTCH3 was found to be overexpressed in COAD, promoting tumor growth as verified through various assays. The model effectively predicted COAD prognosis and patient responses to treatments, underscoring the potential of BCAA pathways as therapeutic targets.

**Conclusion:**

The BRS is instrumental in predicting the prognosis and therapeutic response in COAD, with NOTCH3 playing a significant role in the proliferation, invasion and migration of COAD. These findings suggest that targeting BCAA metabolism and NOTCH3 could advance COAD treatment strategies.

## Introduction

Colon adenocarcinoma (COAD) is among the most prevalent malignancies worldwide, characterized by distinct pathophysiological and epidemiological traits (1). Pathophysiologically, its development is closely linked to genetic predispositions, lifestyle factors such as diet, and chronic inflammation (2). Epidemiological studies have highlighted significant global incidence disparities, closely associated with regional differences, levels of economic development, and lifestyles (3). The complex etiology of colorectal cancer involves genetic mutations, alterations in epigenetic modifications, and the influence of the microenvironment (4). One of the major challenges in treating colorectal cancer lies in its asymptomatic nature in early stages, often leading to delayed diagnoses (5). For advanced-stage patients, achieving desirable outcomes with the most advanced treatments available remains difficult (6). Despite progress in early diagnosis and treatment, managing colorectal cancer continues to pose significant challenges, especially for late-stage patients (7). Thus, researching new therapeutic targets and mechanisms to develop more effective treatment strategies represents a crucial frontier in colorectal cancer research.

Branched-chain amino acids (BCAAs), including leucine, isoleucine, and valine, are essential components of human nutrition, primarily involved in protein synthesis and energy metabolism (8, 9). Abnormalities in BCAA metabolism, observed not only in colorectal cancer but also in various other tumors, suggest this metabolic pathway may be a common regulatory factor in tumor growth and progression (10–12). BCAAs play a pivotal role in supporting tumor cell growth and survival, with their metabolic products meeting the energy needs of tumor cells, promoting protein synthesis, and counteracting cell death (13). Additionally, BCAA metabolism can influence immune cell functionality, altering immune responses within the tumor microenvironment to further promote tumor growth and metastasis. Recent research indicates that abnormalities in BCAA metabolism play a key role in the development and progression of various tumors. Particularly within the tumor microenvironment, changes in BCAA metabolism can regulate tumor cell growth, survival, and invasiveness, thereby impacting tumor progression (14). Therefore, exploring the mechanisms of BCAA metabolism in tumors, especially in colorectal cancer, holds significant value in identifying new therapeutic targets.

In this study, we constructed and validated a BCAA-related predictive model aimed at forecasting the prognosis of COAD patients. Furthermore, we delved into the correlations between our model and factors such as immune infiltration, responses to immunotherapy, characteristics of the tumor microenvironment, and clinical variables. We further elucidated the role of NOTCH3 in COAD proliferation and invasion through experiments such as plate cloning, CCK8, and others.

## Methods

### Data acquisition and processing

Obtain the transcript data of COAD patients from the TCGA and GEO databases. Employ the GSE17538 (15) and GSE39582 (16) datasets for external validation. Acquire single cell sequencing data for COAD patients (GSE166555) (17) from the Tumor Immune Single Cell Hub 2 (TISCH2) (18). Compile a list of 30 BCAA related metabolism genes by summarizing information from reviews and other scholarly articles (19–21).

### Single-cell sequencing analysis

The TISCH2 database is an advanced platform designed to provide comprehensive single-cell transcriptomic data across various types of cancers. It serves as an invaluable resource for researchers aiming to understand the tumor microenvironment at a single-cell resolution. TISCH2 focuses on the detailed characterization of the cellular composition within tumors, highlighting the interactions between tumor cells and the immune system. By offering access to single cell sequencing data, including information on immune cell types and their functional states, TISCH2 facilitates the exploration of immune infiltration in cancer, the discovery of potential immunotherapy targets, and the understanding of tumor heterogeneity. This database represents a significant step forward in precision oncology, enabling the development of more effective, individualized treatment strategies. We obtained differentially expressed genes from the GSE166555 dataset within TISCH2 to screen for valuable genes implicated in COAD progression.

### Prognostic model analysis

Based on the expression profiles of genes intersecting between differentially expressed genes within tumors in GSE166555 and BCAA-related metabolism genes, a BCAA metabolism-related signature (BRS) was constructed using univariate Cox regression and Least Absolute Shrinkage and Selection Operator (Lasso) regression analysis. COAD patients with available survival data were then evenly divided into two groups based on the median value of their risk scores. Subsequently, various algorithms were used to assess the accuracy and reliability of the model’s predictions.

### Immune microenvironment analysis

The single-sample Gene Set Enrichment Analysis (ssGSEA) is a computational method that evaluates the enrichment of gene sets within individual samples, providing a way to derive quantitative scores that reflect the degree of activity or presence of specific biological processes or pathways in each sample. We utilized ssGSEA to calculate the enrichment scores for immune cells and immune functions in COAD samples, enabling us to unveil the detailed composition of immune cells present in the samples. This approach allows for a deeper understanding of the immune dynamics within the context of the disease under study. The ESTIMATE algorithm is employed to assess the abundance of immune cells within the tumor microenvironment (TME) of COAD samples. The IMvigor210 database is used to validate differences in immunotherapy outcomes between model groups.

### Mutation and drug sensitivity analysis

The tumor mutational burden (TMB) is a measure of the number of mutations within the DNA of a tumor, serving as an indicator of tumor aggressiveness and a potential predictor of response to immunotherapy. The correlation between TMB and the BCAA-related predictive model is assessed using the “maftools” R package along with Kaplan-Meier (KM) survival curve analysis. In the analysis of targeted therapeutic drugs, the “pRRophetic” package is utilized to evaluate the half-maximal inhibitory concentration (IC50) of nine common chemotherapy drugs for COAD.

### Patient selection criteria

Inclusion Criteria:1. Age between 18 and 80 years. 2. Pathologically confirmed primary COAD after surgery. 3. Patients who have not received preoperative radiotherapy or chemotherapy. Exclusion Criteria: 1. Patients who have received preoperative radiotherapy or chemotherapy. 2. Patients with other types of malignancies or severe systemic diseases. 3. Patients unable to provide adequate medical history or follow-up data. All samples were frozen at -80°C for long-term storage. All patients signed an informed consent form before specimen collection. This study was approved by the First Affiliated Ethics Committee of Nanjing Medical University.

### Statistical analysis

All analyses were conducted using R version 4.2.2. The significance of differences between groups was assessed using the Chi-squared test for categorical variables and the Wilcoxon rank-sum test for continuous variables. For all tests, a two-sided p-value of less than 0.05 was considered statistically significant. The “survminer” package was used to determine the optimal cutoff value. Cox regression and KM analysis were carried out using the “survival” package. **Supplementary Table 1** records the specific experimental schemes and steps. **Supplementary Table 2** lists the acronyms, definitions, roles of the 30 BCAA-related genes, and the differentially expressed genes from GSE166555.

## Results

### Cell type analysis and BCAA metabolism-related gene screening

**Figure 1:**
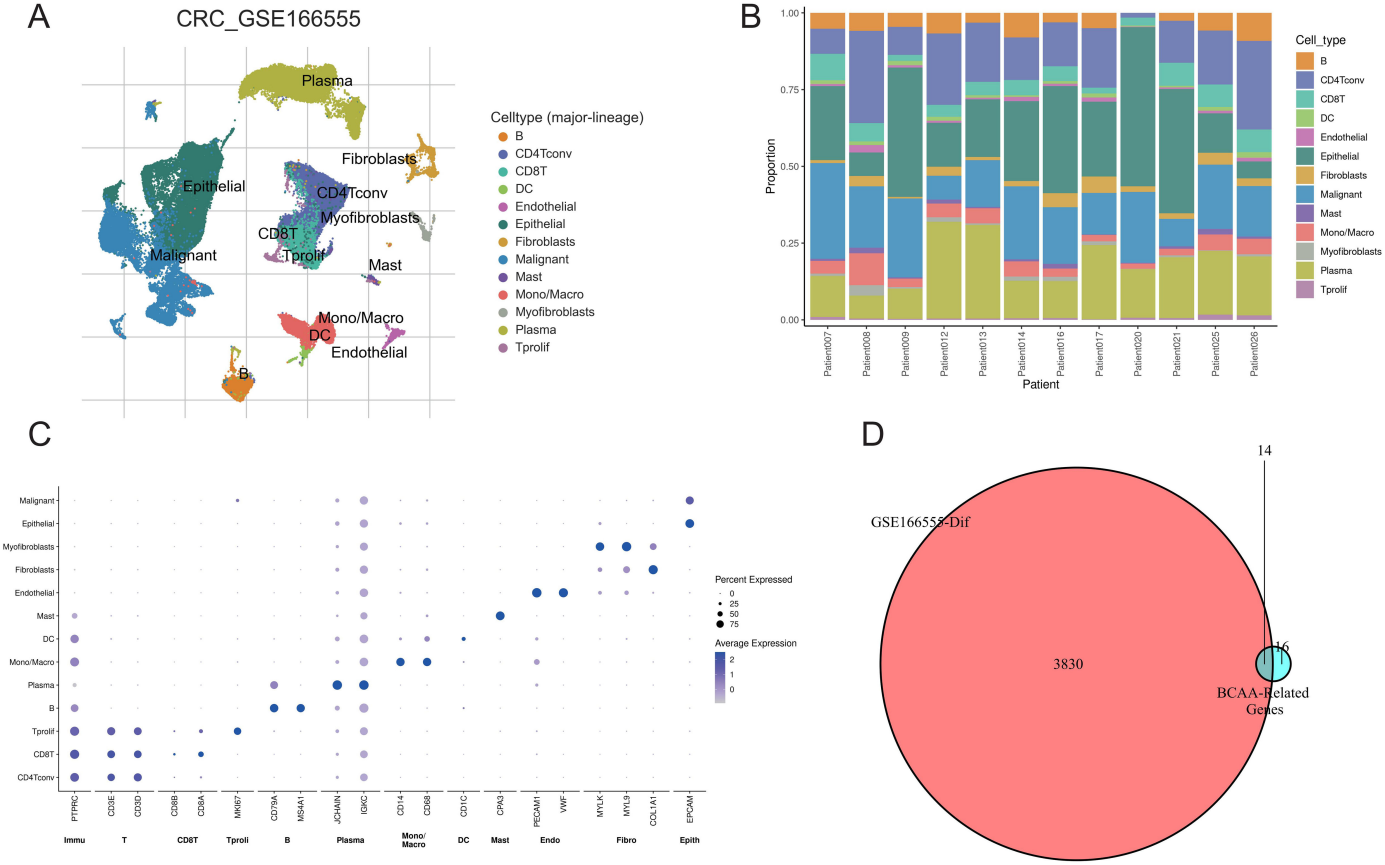
Single-cell analysis and gene screening. **(A)** Distribution of 13 cell types in GSE166555; **(B)** Proportion of different cells in each sample; **(C)** Expression of marker genes for each cell type; **(D)** Screening of differentially expressed BCAA metabolism-related genes.

We conducted a clustering analysis on the GSE166555 dataset using the TISCH2 database, resulting in the identification of co-clusters for 13 distinct cell types (**Figure 1A**). Additionally, we examined the expression patterns of these 13 cell types across different samples (**Figure 1B**). The expression of cell-specific marker genes across various cell types was depicted in **Figure 1C**. Through a review of the literature, we compiled a list of 30 genes related to BCAA metabolism. Subsequently, applying a criterion of an adjusted p-value < 0.001, we identified 3,844 genes from the GSE166555 dataset that showed significant differential expression. Ultimately, via a cross-analysis of the BCAA metabolism-related genes with the differential gene expression library, we pinpointed 14 genes of interest (**Figure 1D**).

### Development and validation of the BCAA metabolism-based prognostic model

**Figure 2:**
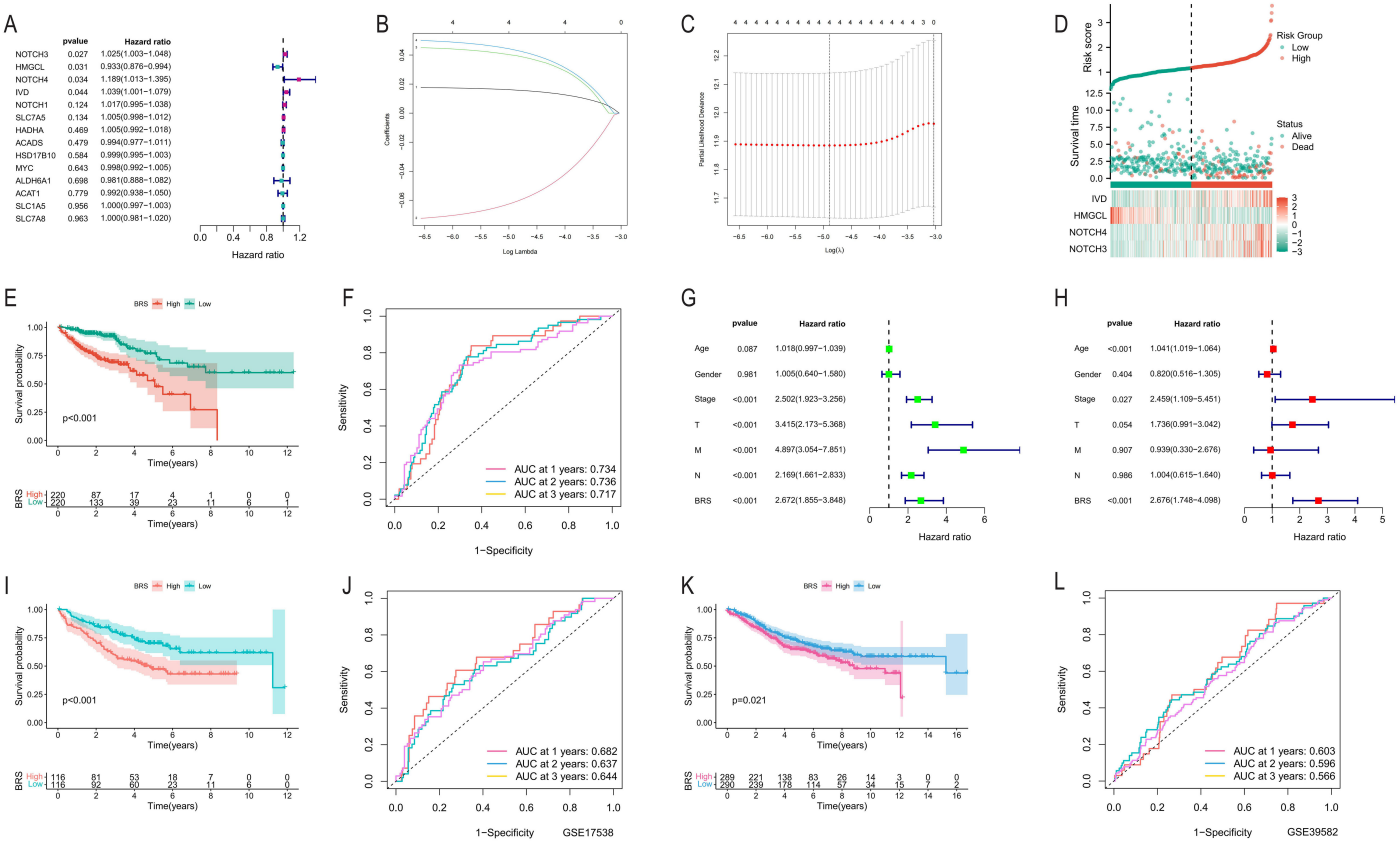
Development and validation of the BCAA metabolism-based prognostic model. **(A)** Univariate Cox regression analysis of 14 intersecting genes; **(B, C)** Application of Lasso analysis to identify prognostic BCAA metabolism-related genes with the minimum lambda value; **(D)** Scatter plot reordering each sample’s risk curve based on BRS and survival status; **(E)** Survival curve differences between high and low BRS groups; **(F)** AUC predictions for the model in the first, second, and third year; **(G, H)** Univariate and multivariate independent prognostic analysis results of the model; **(I, K)** Comparison of KM survival curves between high and low-risk groups in GSE17538 and GSE39582; **(J, L)** AUC predictions for the model in the first, second, and third year in GSE17538 and GSE39582.

We conducted a univariate Cox regression analysis on 14 intersecting genes and identified four significant genes according to a p-value criterion of <0.05 (**Figure 2A**). Subsequently, we utilized LASSO analysis with optimized parameter λ to select four prognostically significant genes for constructing a BCAA metabolism-related predictive model for COAD patients (**Figures 2B, C**). **Figure 2D** illustrates that as the risk score increases, so does the number of deceased patients. KM curves demonstrate that the high-risk group has a poorer prognosis (**Figure 2E**). This predictive model shows good accuracy in forecasting the prognosis of COAD patients at 1, 2, and 3 years, with area under the curve (AUC) values exceeding 0.71 (**Figure 2F**). Univariate and multivariate independent prognostic analyses further confirm the model’s capability as an independent prognostic factor, accurately predicting patient outcomes (**Figures 2G, H**). In external validation cohorts (GSE17538 and GSE39582), high-risk COAD patients exhibited worse clinical outcomes, although the AUC values were lower (**Figures 2I–L**).

### Analysis of immune suppression and tumor microenvironment complexity

**Figure 3:**
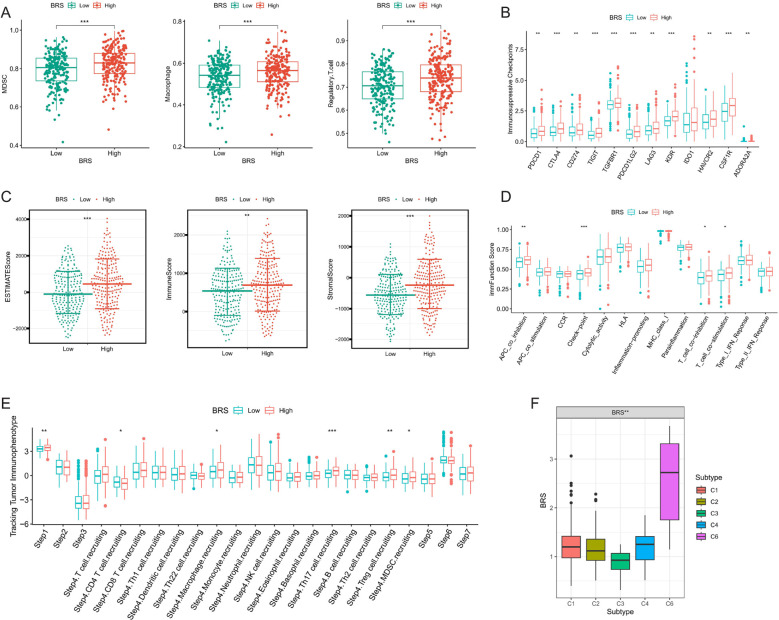
Analysis of immune suppression and tumor microenvironment complexity. **(A)** Differences in the expression of immunosuppressive cells between high and low BRS groups; **(B)** Variations in immunosuppressive checkpoint expression between high and low BRS groups; **(C)** Disparities in tumor microenvironment scores between high and low BRS groups; **(D)** Contrasts in immune function scores between high and low BRS groups; **(E)** Variability in BRS expression across different Tracking Tumor immunophenotypes; **(F)** Differences in BRS expression among various immune subtypes. *P < 0.05, **P < 0.01, ***P < 0.001.

Firstly, we employed the ssGSEA algorithm to assess the infiltration levels of immune suppressive cells (macrophages, myeloid derived suppressor cells (MDSCs), and Regulatory T cells) in each COAD sample. We observed that all three immune suppressive cell types were significantly overexpressed in the high BRS group (**Figure 3A**). Subsequently, our analysis revealed a significant upregulation of most immune suppressive checkpoints in the high BRS group (**Figure 3B**). Within the TME, stromal scores, immune scores, and ESTIMATE scores were notably higher in the high BRS group compared to the control group (**Figure 3C**). Regarding immune function scoring, the high BRS group demonstrated significantly higher APC co-inhibition, T-cell suppression, and checkpoint immunoscore (**Figure 3D**). We then analyzed the anti-cancer immune status across seven stages in COAD patients through the cancer-immunity cycle analysis from the Tracking Tumor Immunophenotype (TIP) database. We found that the stages involving immune suppressive cells were significantly overexpressed in the high BRS group (**Figure 3E**). To further explore the complexity and diversity of the TME in COAD, especially the interactions between tumor cells and the immune system, we analyzed the expression differences of BRS across immune subtypes (C1-C6). The results indicated that BRS expression was significantly higher in C4 and C6 (**Figure 3F**), subtypes characterized by immune exclusion or suppression, potentially indicating a poorer prognosis. This aligns with the research findings presented above.

### TMB and immunotherapy response across BRS groups

Given the potential impact of tumor mutational burden (TMB) on the efficacy of immunotherapy, we further investigated the differences in TMB across various BRS groups. We observed that the expression of TMB was significantly higher in the high BRS group (**Supplementary Figure 1A**). The mutation rate was 184/194 (94.88%) in the low BRS group, compared to 189/193 (97.93%) in the high BRS group (**Supplementary Figures 1B, C**). Additionally, a high BRS was associated with poor prognosis (**Supplementary Figure 1D**). KM survival curves revealed that the best prognosis was observed in patients with Low-TMB+Low-BRS, while the worst prognosis was seen in those with High-TMB+High-BRS (**Supplementary Figure 1E**). To further guide clinical decision-making, we evaluated the differential outcomes of immunotherapy using the IMvigor210 database. The prognosis was poorer in the high BRS group, with a significantly higher proportion of patients experiencing stable disease (SD) or progressive disease (PD) compared to the low BRS group (**Supplementary Figures 1F, G**). By analyzing data from The Cancer Imaging Archive (TCIA), we discovered that CTLA4 might have better therapeutic effects on patients in the low BRS group (**Supplementary Figures 1H–J**).

### Chemotherapy drug sensitivity across BRS groups

To further guide clinical strategy development, we analyzed the differences in IC50 values of nine chemotherapy drugs between BRS groups. Our findings indicate that Sorafenib and Lapatinib exhibit higher IC50 values in the high BRS group, suggesting they may be more effective for patients in the low BRS group (**Supplementary Figures 2A, B**). Conversely, Axitinib, Bleomycin, Pazopanib, Lenalidomide, Imatinib, Nilotinib, and Gefitinib showed higher IC50 values across both groups, indicating that these seven drugs might be better suited for patients with higher BRS (**Supplementary Figures 2C–I**).

### Expression and prognostic features of four modeling genes in COAD

**Figure 4:**
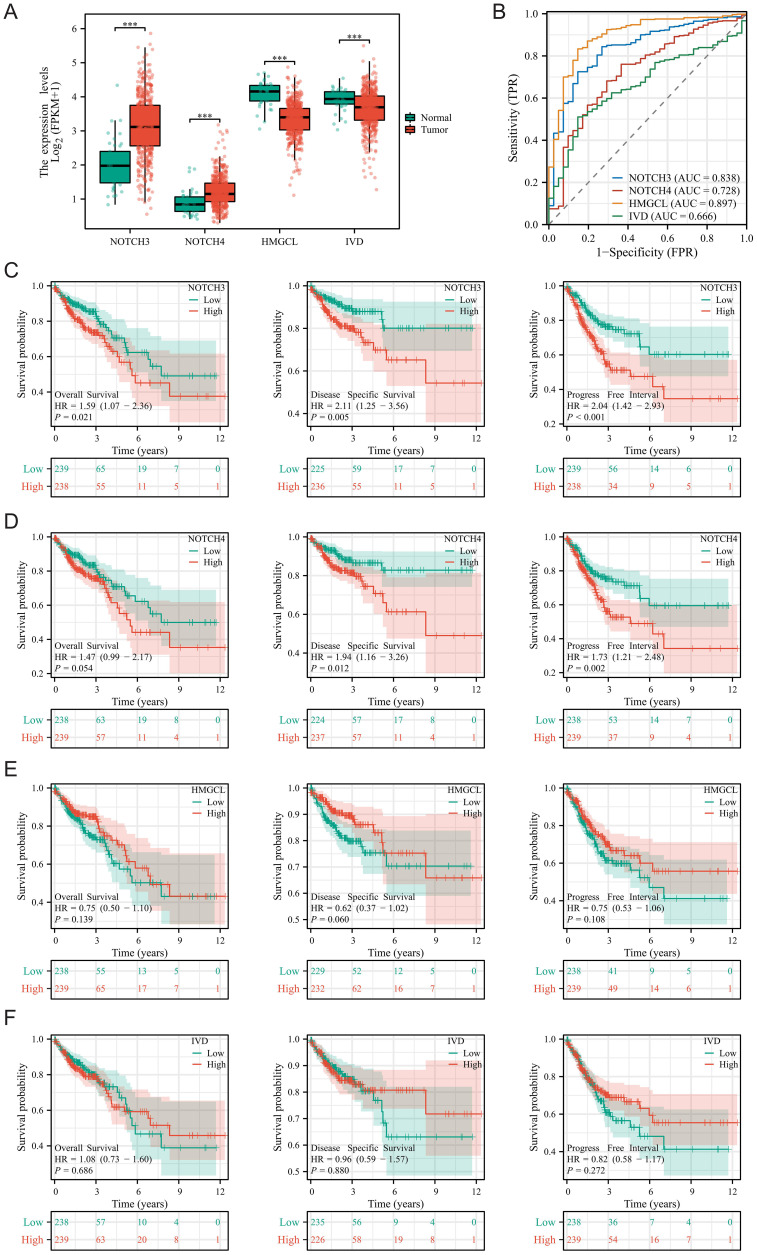
Expression and prognostic features of four modeling genes in COAD. **(A)** Differences in the expression of four modeling genes between tumor tissues and adjacent normal tissues; **(B)** Diagnostic ROC curves for the four modeling genes; **(C–F)** Survival differences for the four modeling genes across Overall Survival, Disease-Specific Survival, and Progression-Free Interval. ***P < 0.001.

We delved deeper into the expression and prognostic characteristics of four modeling genes (NOTCH3, NOTCH4, IVD, and HMGCL) in COAD. **Figure 4A** showed that NOTCH3 and NOTCH4 were significantly overexpressed in tumor patients, whereas IVD and HMGCL showed the opposite trend. Diagnostic ROC curves revealed that HMGCL had the highest AUC, followed by NOTCH3 (**Figure 4B**). Subsequently, we stratified COAD patients into high and low expression groups based on the median value of gene expression and analyzed the survival differences in overall survival (OS), disease-specific survival (DSS), and progression-free interval (PFI) between these groups. KM curves indicated that patients in the high expression groups of NOTCH3 and NOTCH4 are associated with poor prognosis, while there were no significant survival differences observed between the HMGCL and IVD groups (**Figures 4C–F**).

**Supplementary Figure 3** illustrated the expression levels and prognostic significance of the four modeling genes (NOTCH3, NOTCH4, HMGCL, IVD) across various COAD conditions and outcomes. **Supplementary Figure 3A** compared gene expression across normal tissue versus early (Stage I&II) and late-stage (Stage III&IV) COAD, showing notably higher expression of NOTCH3 and NOTCH4 in all stages of cancer compared to normal, while HMGCL and IVD were predominantly lower in cancer stages. **Supplementary Figure 3B** focused on tumor size (T stage), demonstrating similar trends of expression among the genes across normal tissue, T1&T2, and T3&T4 categories. **Supplementary Figures 3C, D** depicted the expression patterns relative to lymph node involvement (N0, N1&N2) and the presence of distant metastasis (M0, M1), respectively, highlighting a consistent overexpression of NOTCH genes in the presence of metastasis. **Supplementary Figures 3E, F** addressed the expression levels in the context of perineural invasion and OS, respectively, with noticeable differences in gene expression between patients with and without perineural invasion and between alive and deceased patients, underscoring the prognostic potential of these genes. Finally, **Supplementary Figures 3G, H** showed the expression of genes related to DSS and PFI, respectively, where differences in expression correlate with patient outcomes, further emphasizing the relevance of these genes in predicting prognosis in COAD. Overall, the most significant differences in the expression of NOTCH3 across various clinical aspects of COAD suggest its potential as a biomarker for disease progression and prognosis. Therefore, we chose to further explore the role of NOTCH3 in COAD proliferation and invasion.

### Validation of NOTCH3 expression levels in COAD

**Figure 5:**
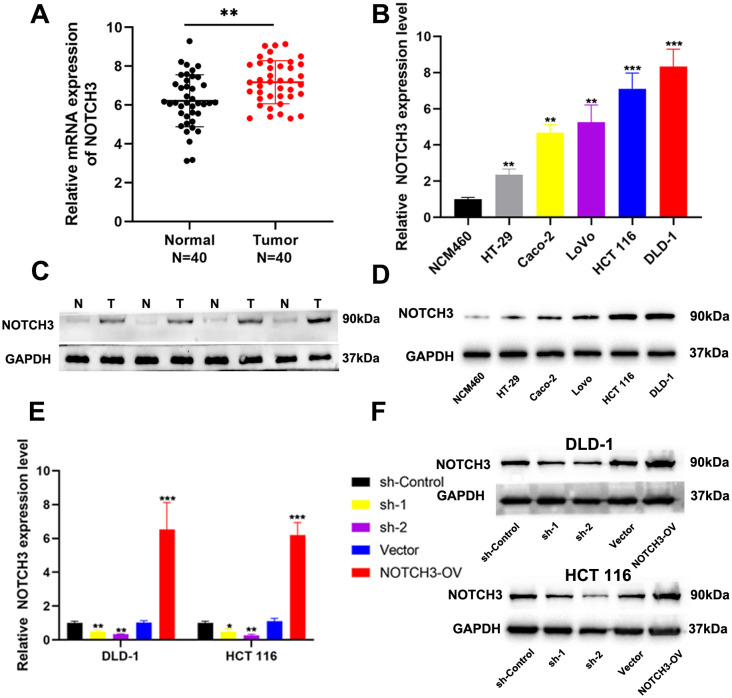
Expression of NOTCH3 in tumor tissues and cell lines. **(A)** The mRNA expression of NOTCH3 in 40 pairs of CRC tissues and adjacent normal tissues; **(B)** The mRNA expression of NOTCH3 in CRC cell lines and NCM460; **(C, D)** The protein level of NOTCH3 in cell lines and tissues by western blot assay; **(E, F)** NOTCH3 knockdown and overexpression efficiencies in DLD-1 and HCT 116 cells were determined by RT-PCR and Western blotting. (*P < 0.05, **P < 0.01, ***P<0.001).

To further investigate the role of NOTCH3 in the progression of COAD, we initially analyzed the mRNA expression of NOTCH3 in COAD tissues compared with adjacent normal tissues and various COAD cell lines. Our findings revealed a statistically significant increase in NOTCH3 mRNA expression in tumor tissues and COAD cell lines (**Figures 5A, B**). Subsequently, we utilized Western blot analyses to assess NOTCH3 protein expression levels in COAD tissues, adjacent normal tissues, and several cell lines. These analyses confirmed that NOTCH3 protein levels were significantly elevated in tumor tissues and COAD cell lines (**Figures 5C, D**), underscoring its potential role in tumorigenesis. To further explore the effects of NOTCH3 on COAD proliferation and invasion, we selected two cell lines with the highest NOTCH3 expression levels, HCT 116 and DLD-1, for knockdown and overexpression studies. Using qRT-PCR and Western blot assays, we demonstrate that lentiviral vector transfection of interfering and overexpressed sequences can significantly modulate NOTCH3 mRNA and protein expression in both cell lines (**Figures 5E, F**).

### *In vitro* effects of NOTCH3 knockdown, overexpression, and recovery assays on COAD growth and metastasis

**Figure 6:**
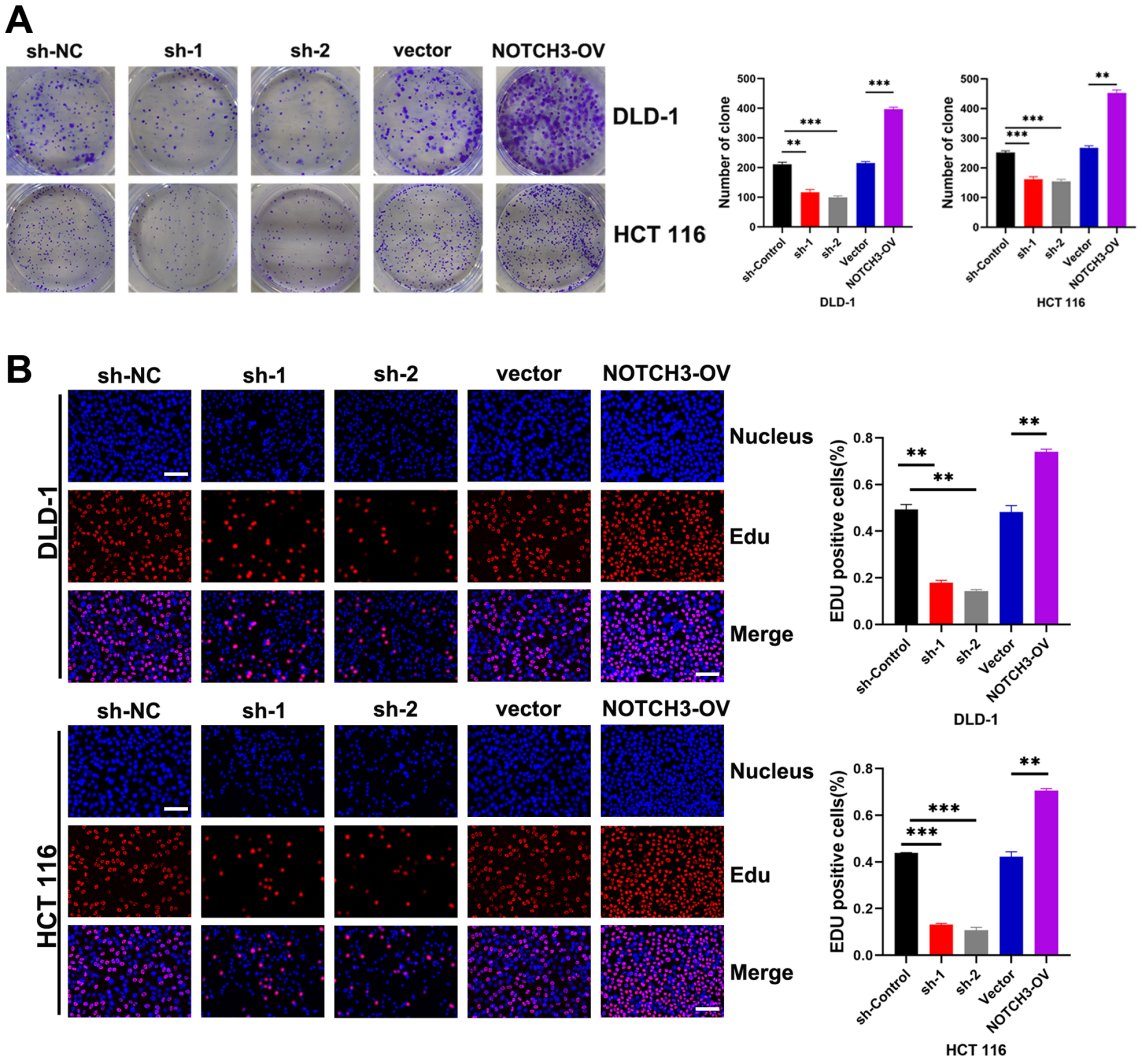
NOTCH3 promotes the proliferation of colon cancer cells. **(A)** Effects of knockdown and overexpression of NOTCH3 on cell colony formation ability in DLD-1 and HCT 116 cells; **(B)** EdU assay shows the effect of knocking down or overexpressing NOTCH3 on the proliferation ability of DLD-1 and HCT 116 cells in the treatment group and control group respectively (scale bar:50μm). (**P < 0.01, ***P<0.001).

**Figure 7:**
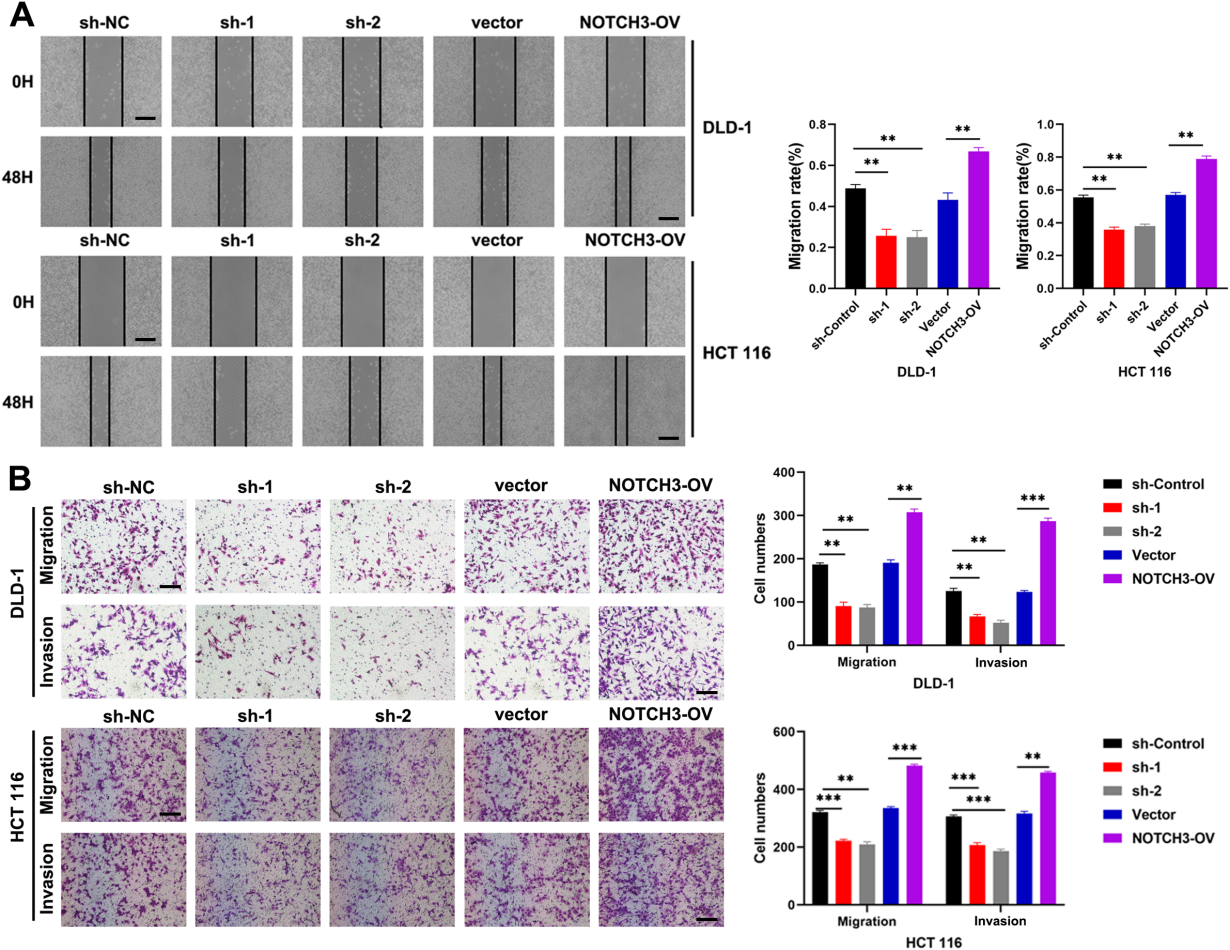
NOTCH3 promotes CRC migration and invasion *in vitro*. **(A)** The wound healing assay was used to detect the effect on cell migration ability after knocking down or overexpressing NOTCH3 in DLD-1 and HCT 116 cells (scale bar: 100μm); **(B)** Transwell assay was used to detect changes in the migration and invasion numbers of colon cancer cells after knocking down or overexpressing NOTCH3 (scale bar: 100μm) (**P < 0.01, ***P<0.001).

Through colony formation assays, we observed that the proliferation of COAD cells was significantly inhibited in the NOTCH3-knockdown groups (sh1 and sh2) in DLD-1 and HCT 116 cell lines, while proliferation was significantly enhanced in the NOTCH3-overexpression (NOTCH3-OV) group (**Figure 6A**). Similarly, EdU incorporation assays revealed that NOTCH3 knockdown markedly suppressed the proliferative capacity of COAD cells in both DLD-1 and HCT 116 lines, whereas NOTCH3 overexpression promoted this capacity (**Figure 6B**). Wound healing assays demonstrated that, after 48 hours, the migration distance of DLD-1 and HCT-116 cells in the NOTCH3-knockdown groups (sh1 and sh2) was significantly reduced compared to the control group, whereas the NOTCH3-OV group exhibited notably enhanced migration (**Figure 7A**). Furthermore, Transwell assays showed that the invasion and migration abilities of DLD-1 and HCT-116 cells were significantly curtailed in the NOTCH3-knockdown groups, while these abilities were increased in the NOTCH3-OV group (**Figure 7B**). Subsequent experiments, including colony formation and EdU assays, demonstrated that supplementing branched-chain ketoacids (BCKAs) restored proliferation capabilities in DLD-1 and HCT 116 cell lines following NOTCH3 knockdown (**Supplementary Figures 4A–C**). Additionally, wound healing and Transwell assays indicated that BCKA supplementation reinstated the invasive and migratory capacities of these cells (**Supplementary Figures 4D, E**). These findings collectively indicate that NOTCH3 plays a critical role in the proliferation, migration, and invasion of COAD cells, and its expression is positively correlated with these oncogenic processes.

### *In vivo* effects of NOTCH3 knockdown and overexpression on COAD growth and metastasis

**Figure 8:**
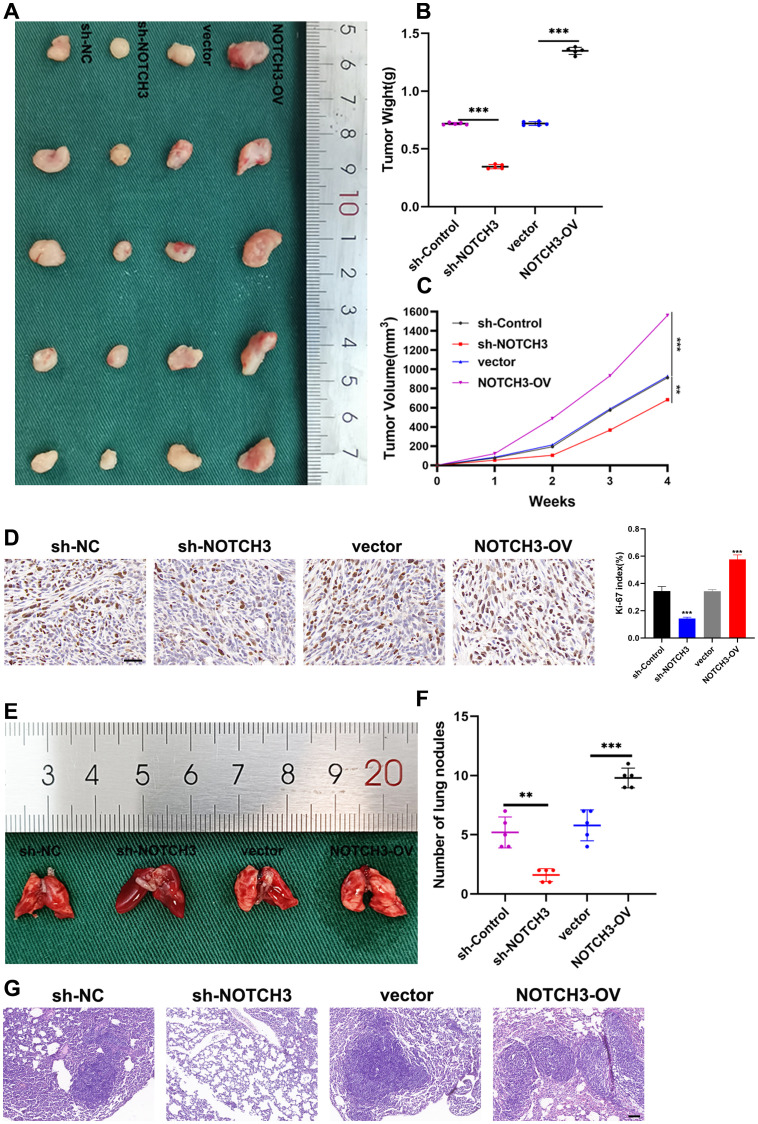
NOTCH3 promotes colon cancer cell proliferation and lung metastasis *in vivo*. **(A)** Size of subcutaneous transplanted tumors in mice with knockdown or overexpression of NOTCH3 and control group; **(B)** After 4 weeks, the mice were sacrificed to obtain tumors from different treatment groups and the final weights were measured for analysis; **(C)** After inoculating cells subcutaneously into mice, observe the growth of subcutaneous tumors in nude mice every week and measure the long and transverse diameters of subcutaneous tumors in tumor-bearing mice, estimate tumor volume and draw a growth curve; **(D)** Immunohistochemistry was used to detect the expression of the proliferation marker Ki-67 in subcutaneous xenograft tumors finally obtained after four groups of treatments (scale bar: 50μm); **(E)** Four groups of tumor cells with different treatments were injected into the tail vein of nude mice to establish a lung metastasis model, and the fresh lung tissue obtained after the mice were sacrificed 4 weeks later; **(F)** The number of lung metastases finally obtained after four groups of treatments; **(G)** Representative images of HE staining of mouse lung metastases showing the cross-sectional area of localized lung metastases (scale bar: 100μm) (**P < 0.01, ***P<0.001).

We further assessed the impact of NOTCH3 knockdown and overexpression on tumor growth *in vivo* using a subcutaneous tumor formation assay in nude mice. The results demonstrated that NOTCH3 overexpression led to increased tumor growth, whereas its knockdown resulted in reduced tumor development (**Figures 8A–C**). To evaluate cellular proliferation, we conducted immunohistochemical staining for Ki-67, a well-established proliferation marker, in tumor tissues from the mice. The expression of Ki-67 was markedly lower in the sh-NOTCH3 group compared to the control, indicating a decrease in the proliferative capacity of tumor cells with suppressed NOTCH3 expression *in vivo*. Conversely, Ki-67 staining intensified following NOTCH3 overexpression, suggesting enhanced proliferative activity (**Figure 8D**). Additionally, we explored the effects of NOTCH3 knockdown and overexpression on tumor metastasis using a lung metastasis model in nude mice. NOTCH3 overexpression significantly increased the number of lung metastases, while its knockdown led to a reduction in metastatic spread (**Figures 8E, F**). HE staining revealed that NOTCH3 knockdown notably decreased the cross-sectional area and size of lung metastases, whereas NOTCH3 overexpression increased these parameters, indicating an augmentation of metastatic potential (**Figure 8G**).

## Discussion

COAD continues to pose significant global public health challenges, characterized by its high incidence and mortality rates (22). Epidemiologically, COAD ranks as the third most diagnosed cancer and the second leading cause of cancer-related deaths globally, reflecting its substantial burden on healthcare systems (23). The disease’s etiology is multifaceted, involving a complex interplay of genetic predispositions, environmental factors, and lifestyle choices such as diet, physical inactivity, and smoking (24). Pathophysiologically, COAD develops from precancerous lesions in a well-documented sequence of genetic and epigenetic alterations, leading to the activation of oncogenes and the inactivation of tumor suppressor genes. Despite advances in screening, surgical techniques, and adjuvant therapies, the treatment of COAD faces significant challenges. These include the development of resistance to conventional chemotherapeutic agents, recurrence after surgery, and the limited efficacy of current treatments in advanced-stage disease (25). Therefore, a deeper understanding of COAD’s pathophysiological characteristics is crucial for developing more effective and targeted therapeutic approaches, ultimately aiming to improve patient outcomes and reduce the disease’s overall burden.

The metabolism of BCAAs—leucine, isoleucine, and valine—has emerged as a critical regulatory factor in the pathogenesis and progression of various cancers, including COAD (26, 27). BCAAs extend beyond their fundamental roles in protein synthesis to modulate signal transduction pathways that regulate cell growth, survival, and differentiation (28). Alterations in BCAA metabolism contribute to tumor growth by providing necessary energy and biosynthetic precursors for rapid cancer cell proliferation and invasion (29). Moreover, dysregulated BCAA metabolism influences the TME, affecting immune cell function and contributing to immune evasion mechanisms.

Our study introduces a predictive model specifically focused on BCAA metabolism in COAD. By analyzing gene expression differences related to BCAA metabolism among various patient groups, our model elucidates the complex relationship between BCAA metabolism and COAD progression. This specificity highlights the potential of targeting BCAA metabolic pathways as a novel therapeutic strategy in COAD treatment, providing refined insights that are distinct from other cancer types. The integration of comprehensive genomic data with clinical outcomes enables a nuanced understanding of how BCAA metabolism correlates with disease progression and patient prognosis.

Build the prediction model of BCAA metabolism genes mainly includes: IVD, HMGCL, NOTCH3 and NOTCH4. IVD and HMGCL are pivotal in the catabolism of leucine, affecting energy production and lipid metabolism, which are vital for cancer cell viability. NOTCH3 and NOTCH4, recognized for their roles in cell fate determination, have been found to significantly influence amino acid metabolism and cellular energetics in tumors. The downregulation of HMGCL and IVD in COAD tumors compared to normal tissues could be indicative of a metabolic shift that favors anabolic processes over catabolic ones in cancer cells. By reducing the expression of enzymes involved in leucine degradation, tumor cells might conserve leucine and other BCAAs to support biosynthetic demands, such as protein synthesis, which is essential for rapid cell proliferation. The increased expression of NOTCH3 and NOTCH4 in tumor tissues can be linked to their role in promoting cellular proliferation and survival, common features of tumorigenesis. In cancer, including COAD, BCAA metabolism is often dysregulated, contributing to tumor growth and progression by supporting biosynthesis and influencing the TME. NOTCH3 and NOTCH4 regulate genes involved in BCAA catabolism, thereby affecting the TME and cancer cell metabolism. For example, NOTCH signaling can upregulate BCAT1 and BCAT2, key enzymes in BCAA metabolism (11). This interaction underlines the relevance of including NOTCH3 and NOTCH4 in our analysis, as alterations in these pathways could influence COAD progression and patient outcomes. Additionally, altered BCAA metabolism in pancreatic cancer has been linked with upregulated levels of NOTCH3 and NOTCH4, suggesting their involvement in metabolic adaptations seen in this disease (30). The cross-talk between NOTCH signaling and mTOR pathways, influenced by BCAA levels, suggests that NOTCH could affect cancer metabolism and growth indirectly through BCAA-mediated mTOR activation (12). This connection underlines the potential regulatory influence of NOTCH genes on crucial metabolic pathways in cancerous tissues, highlighting the potential of targeting such interactions for therapeutic benefits.

Particularly, NOTCH3 is profoundly overexpressed in COAD and correlates strongly with tumor proliferation, invasion, and migration, suggesting its potential as a biomarker and therapeutic target. Given these insights, further investigation into the specific roles of NOTCH3 in COAD and its therapeutic potential is imperative. NOTCH3, a member of the Notch receptor family, plays a multifaceted role in tumorigenesis, influencing various aspects of cancer biology including cell proliferation, differentiation, angiogenesis, and apoptosis (31, 32). Its role extends beyond the cellular level to modulate the immune landscape within the TME, impacting immune surveillance and cancer cells’ ability to evade immune destruction (33, 34). In COAD, our findings highlight NOTCH3’s significant differential expression and its correlation with adverse patient outcomes, underscoring its potential as a biomarker for disease progression and as a target for therapeutic intervention. The mechanism through which NOTCH3 contributes to COAD’s pathophysiology involves it signaling pathway that, when dysregulated, leads to the aberrant activation of downstream target genes responsible for cellular proliferation and survival. This can result in the unchecked growth of tumor cells and the suppression of cell death pathways. Additionally, NOTCH3’s interaction with the TME, particularly its influence on angiogenesis and the suppression of anti-tumor immune responses, facilitates tumor growth and metastasis (35, 36). For instance, NOTCH3 signaling can lead to the recruitment of immunosuppressive cell populations or the secretion of cytokines that dampen the effectiveness of the immune response against the tumor (37, 38). In light of our findings, further research into NOTCH3’s specific roles in COAD and its potential as a therapeutic target is warranted. Understanding the nuances of NOTCH3 signaling in COAD could lead to novel approaches to treat this malignancy, potentially improving outcomes for patients with this challenging disease.

We acknowledged the importance of discussing the limitations and suggesting future directions. Recognizing our study’s limitations was crucial for accurately interpreting its results and guiding subsequent research. A significant constraint is our sample size, which, despite including a diverse array of *in vitro* and *in vivo* samples, may not fully represent the heterogeneity of COAD. This could limit how our findings apply to the wider COAD population, where larger cohorts may unveil more about NOTCH3’s role across COAD’s subtypes and stages. The retrospective aspect of analyzing clinical data and outcomes also poses challenges, such as selection biases and dependency on the availability and accuracy of medical records. This aspect may weaken the strength of the correlations between NOTCH3 expression and patient outcomes, suggesting that prospective studies could provide more definitive evidence of causality. Moreover, the applicability of our findings to clinical practice must be considered cautiously. Although our research offers significant insights into NOTCH3’s involvement in COAD, real-world application demands acknowledgment of patient diversity and the intricacies of tumor biology. Verifying how NOTCH3 affects COAD progression and metastasis across a wider clinical spectrum and various genetic contexts is essential for a comprehensive understanding. To address these limitations, future research should involve larger, multi-center, and prospective studies to validate our findings and provide stronger evidence of causality. Additionally, further investigation into the molecular mechanisms of NOTCH3 and its potential as a therapeutic target, as well as exploring other metabolic pathways and integrating multi-omics approaches, is essential.

## Conclusion

Our study constructed a BCAA metabolism-related predictive model for COAD, unveiling significant insights into the disease’s progression. Among the analyzed genes, NOTCH3 stood out for its elevated expression linked to advanced disease stages and poorer outcomes, highlighting its oncogenic role in COAD. Through *in vitro* and *in vivo* validations, we demonstrated NOTCH3’s contribution to tumor proliferation and invasion, positioning it as a potential therapeutic target. This research underscores the importance of metabolic pathways and specific molecular targets, like NOTCH3, in developing effective treatments for COAD.

## Data Availability

The original contributions presented in the study are included in the article/**Supplementary Material**. Further inquiries can be directed to the corresponding author.

## Supplementary material

Supplementary Figure 1 TMB and immunotherapy response across BRS groups. **(A)** Differences in Tumor Mutational Burden (TMB) expression between high and low BRS groups; **(B, C)** Detailed waterfall plots illustrating mutation profiles in high and low BRS groups; **(D)** Survival differences between groups with high and low TMB; **(E)** Survival curves comparing TMB combined with BRS stratifications; **(F)** Survival differences across high and low BRS groups in the IMvigor210 dataset; **(G)** Variations in immunotherapy response rates between high and low BRS groups; **(H–J)** Differences in treatment outcomes with CTLA4 and/or PD1 inhibitors between high and low BRS groups.

Supplementary Figure 2 Chemotherapy drug sensitivity across BRS groups. **(A–I)** IC50 differences for common chemotherapy drugs in high and low BRS groups in COAD.

Supplementary Figure 3 Differential expression and prognostic impact of four modeling genes. **(A-E)** Differences in the expression of modeling genes in different clinical variables (**A**: Pathological stage; **B**: T stage; **C**: Lymph node involvement; **D**: Metastasis; **E**:Perineural invasion) **(F–H)** Correlation with survival outcomes (OS, DSS, PFI). *p<0.05, **p<0.01, ***p<0.001 indicate statistical significance.

Supplementary Figure 4 Supplementation of BCKAs can restore the effect of NOTCH3 knockdown on the function of colon cancer cells. **(A)** Effect of adding BCKAs to NOTCH3-knockdown DLD-1 and HCT 116 cells on colony formation ability. **(B, C)** EdU experiment showed the proliferation ability of DLD-1 and HCT 116 cells in the blank control group and the treatment group (scale: 50μm). **(D)** The wound healing assay was used to examine the effect of BCKAs supplementation on cell migration after knockdown of NOTCH3 in DLD-1 and HCT 116 cells (scale: 100μm). **(E)** Transwell assay was used to detect changes in the number of colon cancer cell migration and invasion after knockdown of NOTCH3 followed by BCKAs supplementation (ruler: 100μm) (ns P>0.05, * P < 0.01).
