# Supplementary figures and images for: Exploiting branched-chain amino acid metabolism and NOTCH3 expression to predict and target colorectal cancer progression
Source: Front Immunol. 2024 Sep 2;15:1430352. doi: 10.3389/fimmu.2024.1430352 (PMC11402679; doi:10.3389/fimmu.2024.1430352)

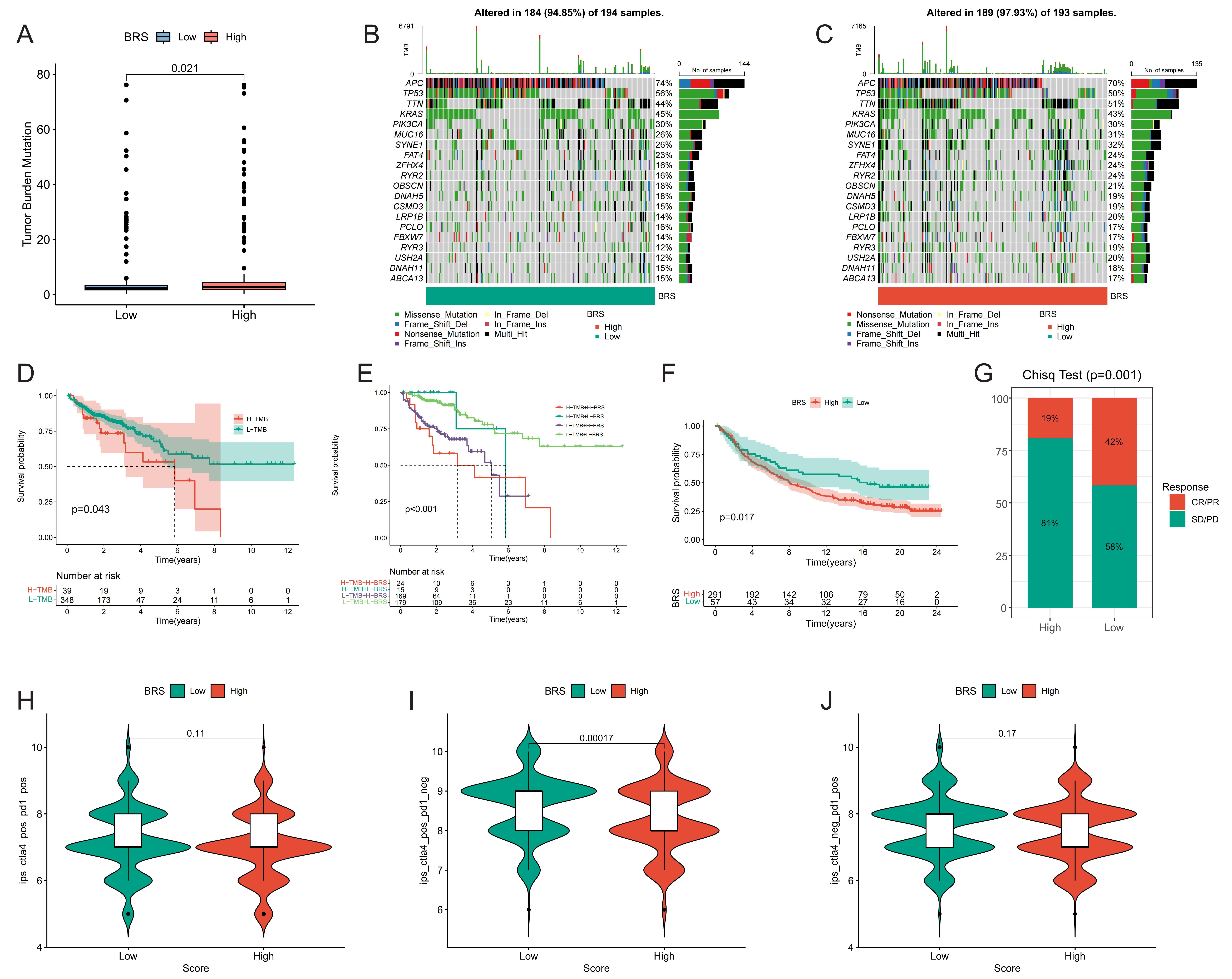

Supplement: Supplementary Figure 1 — TMB and immunotherapy response across BRS groups. (A) Differences in Tumor Mutational Burden (TMB) expression between high and low BRS groups; (B, C) Detailed waterfall plots illustrating mutation profiles in high and low BRS groups; (D) Survival differences between groups with high and low TMB; (E) Survival curves comparing TMB combined with BRS stratifications; (F) Survival differences across high and low BRS groups in the IMvigor210 dataset; (G) Variations in immunotherapy response rates between high and low BRS groups; (H–J) Differences in treatment outcomes with CTLA4 and/or PD1 inhibitors between high and low BRS groups. [file Image1.jpeg]

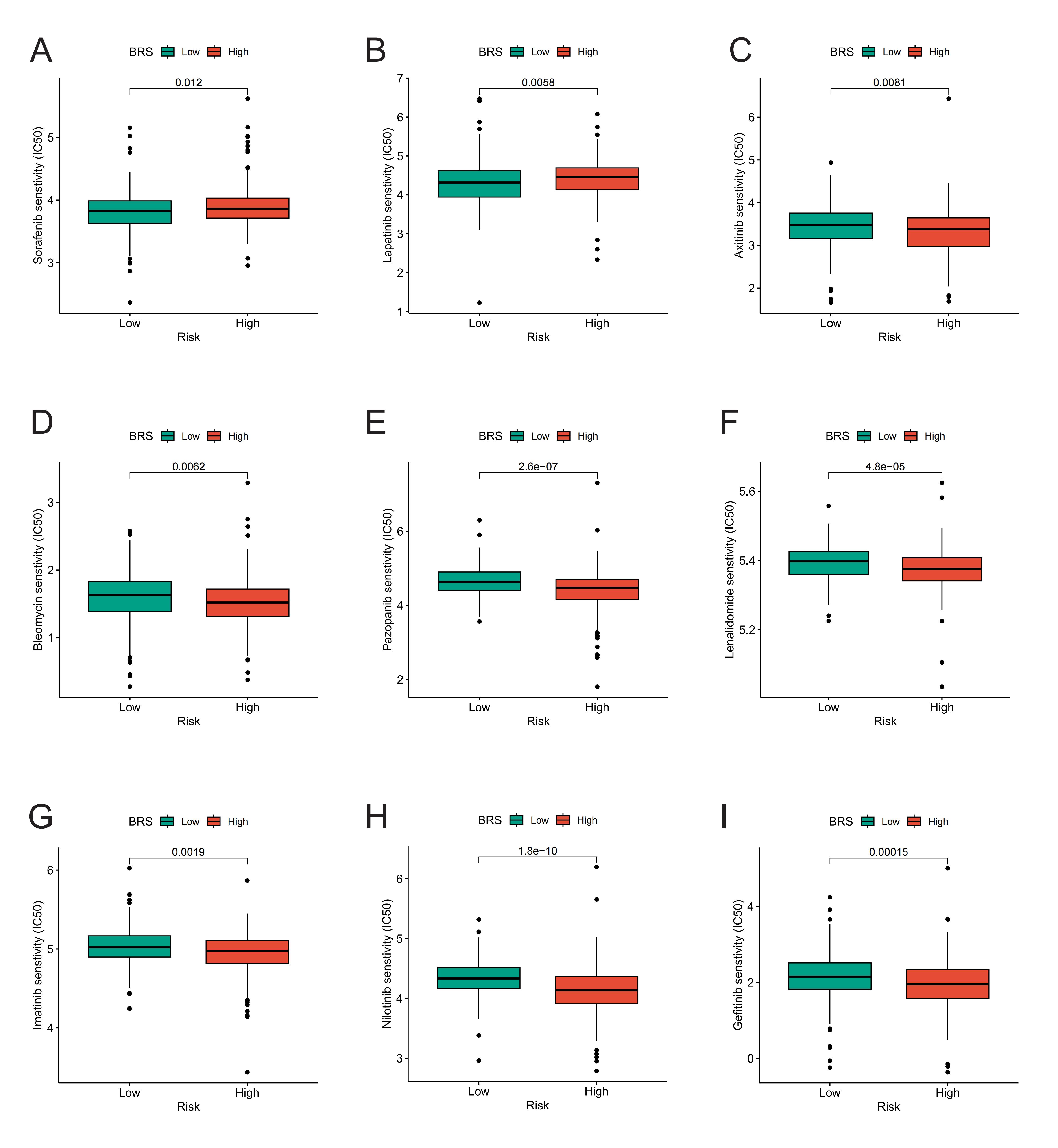

Supplement: Supplementary Figure 2 — Chemotherapy drug sensitivity across BRS groups. (A–I) IC50 differences for common chemotherapy drugs in high and low BRS groups in COAD. [file Image2.jpeg]

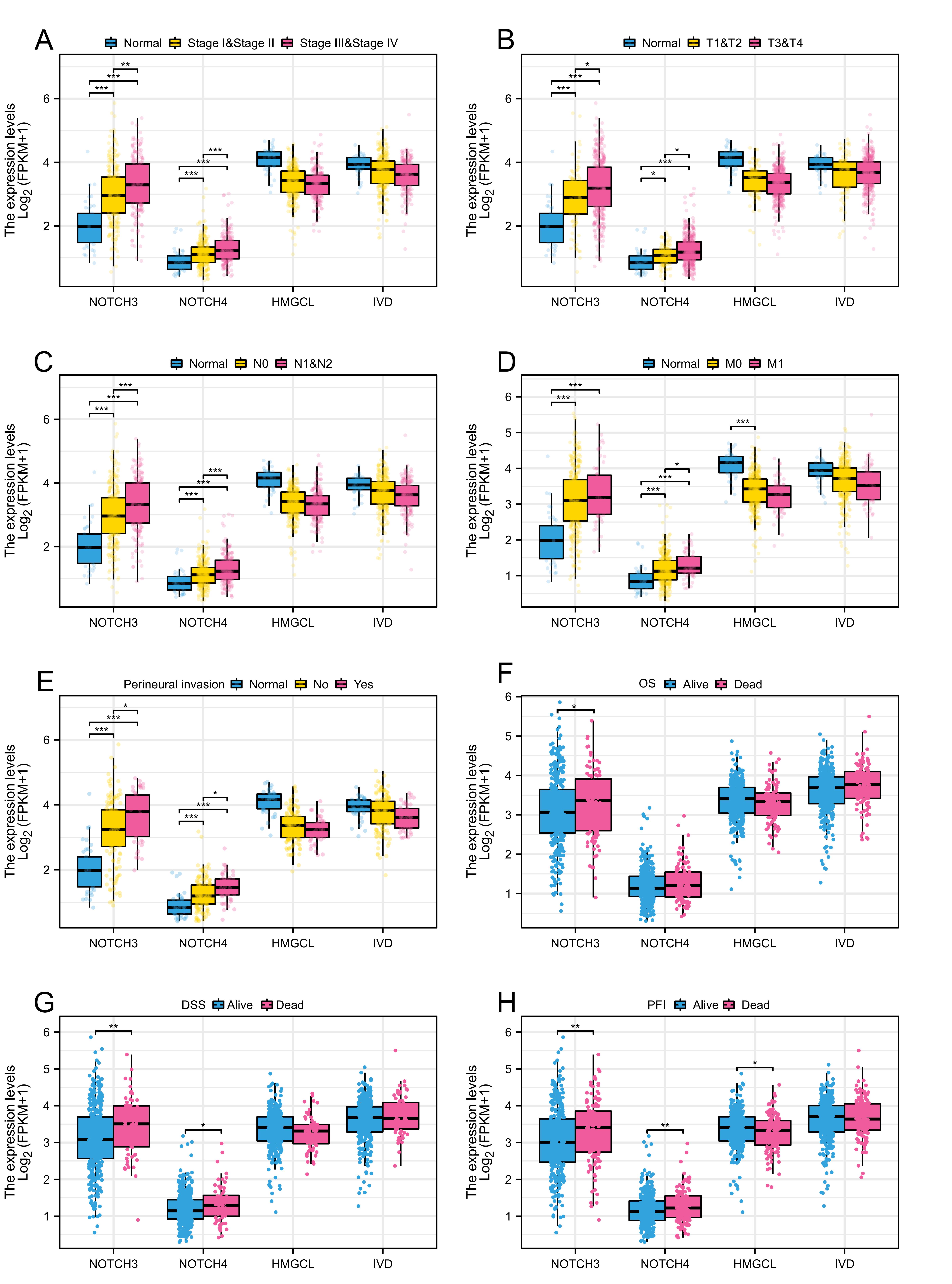

Supplement: Supplementary Figure 3 — Differential expression and prognostic impact of four modeling genes. (A-E) Differences in the expression of modeling genes in different clinical variables (A: Pathological stage; B: T stage; C: Lymph node involvement; D: Metastasis; E:Perineural invasion) (F–H) Correlation with survival outcomes (OS, DSS, PFI). *p<0.05, **p<0.01, ***p<0.001 indicate statistical significance. [file Image3.jpeg]

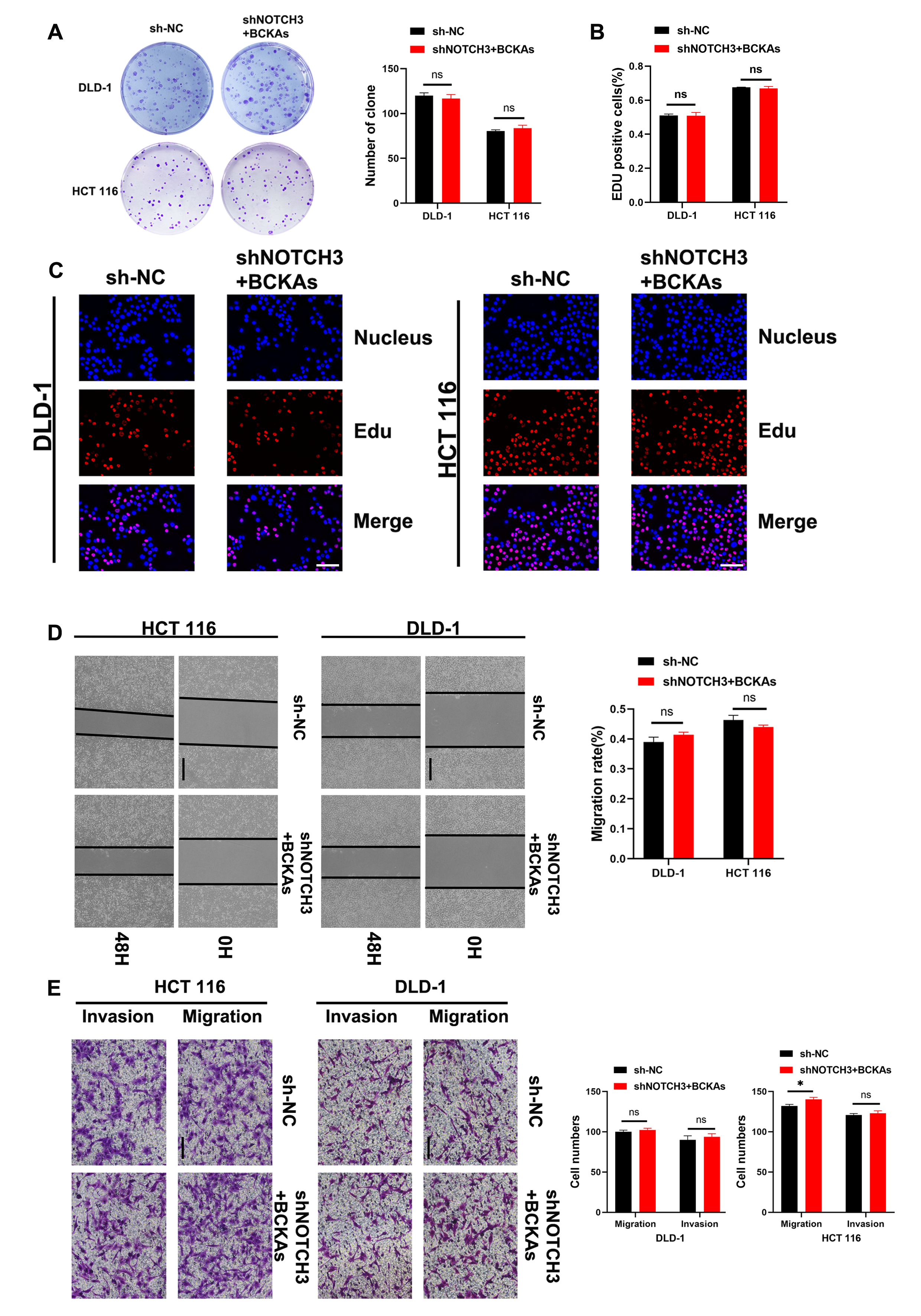

Supplement: Supplementary Figure 4 — Supplementation of BCKAs can restore the effect of NOTCH3 knockdown on the function of colon cancer cells. (A) Effect of adding BCKAs to NOTCH3-knockdown DLD-1 and HCT 116 cells on colony formation ability. (B, C) EdU experiment showed the proliferation ability of DLD-1 and HCT 116 cells in the blank control group and the treatment group (scale: 50μm). (D) The wound healing assay was used to examine the effect of BCKAs supplementation on cell migration after knockdown of NOTCH3 in DLD-1 and HCT 116 cells (scale: 100μm). (E) Transwell assay was used to detect changes in the number of colon cancer cell migration and invasion after knockdown of NOTCH3 followed by BCKAs supplementation (ruler: 100μm) (ns P>0.05, * P < 0.01). [file Image4.tif]

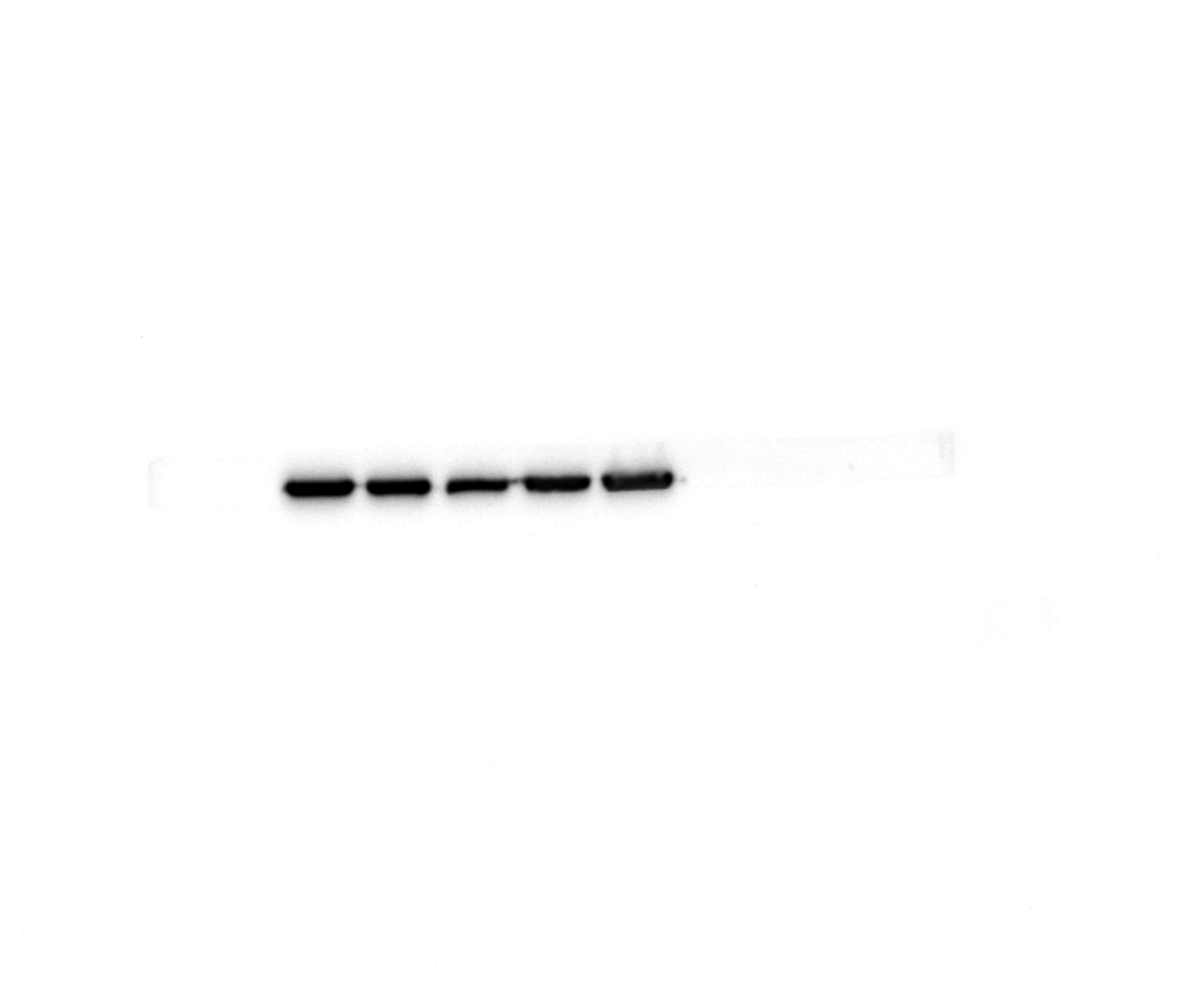

Supplement: Supplementary file 5 [file DataSheet1.zip › WB/敲低过表达/GAP.Tif]

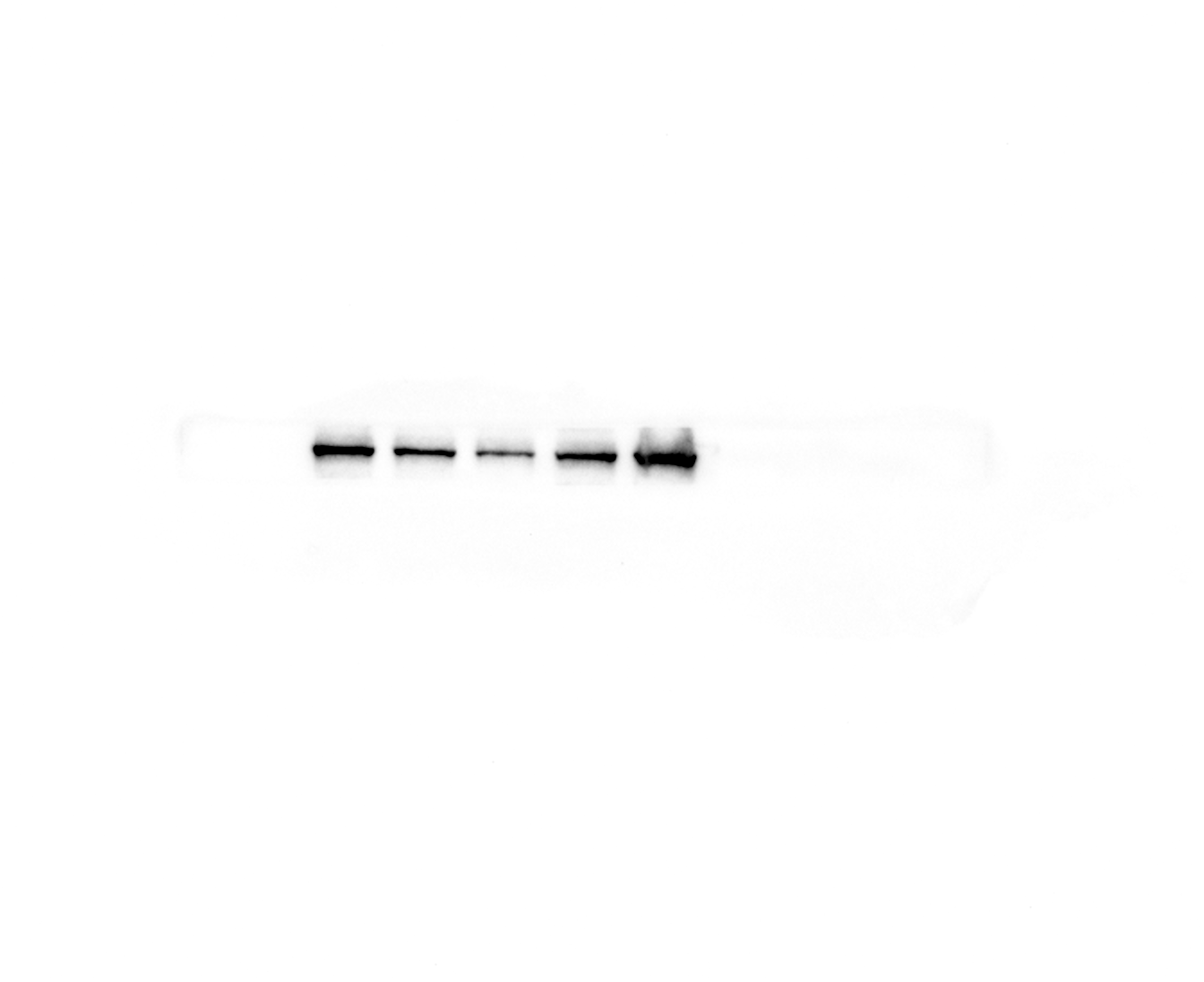

Supplement: Supplementary file 5 [file DataSheet1.zip › WB/敲低过表达/NOTCH3.Tif]

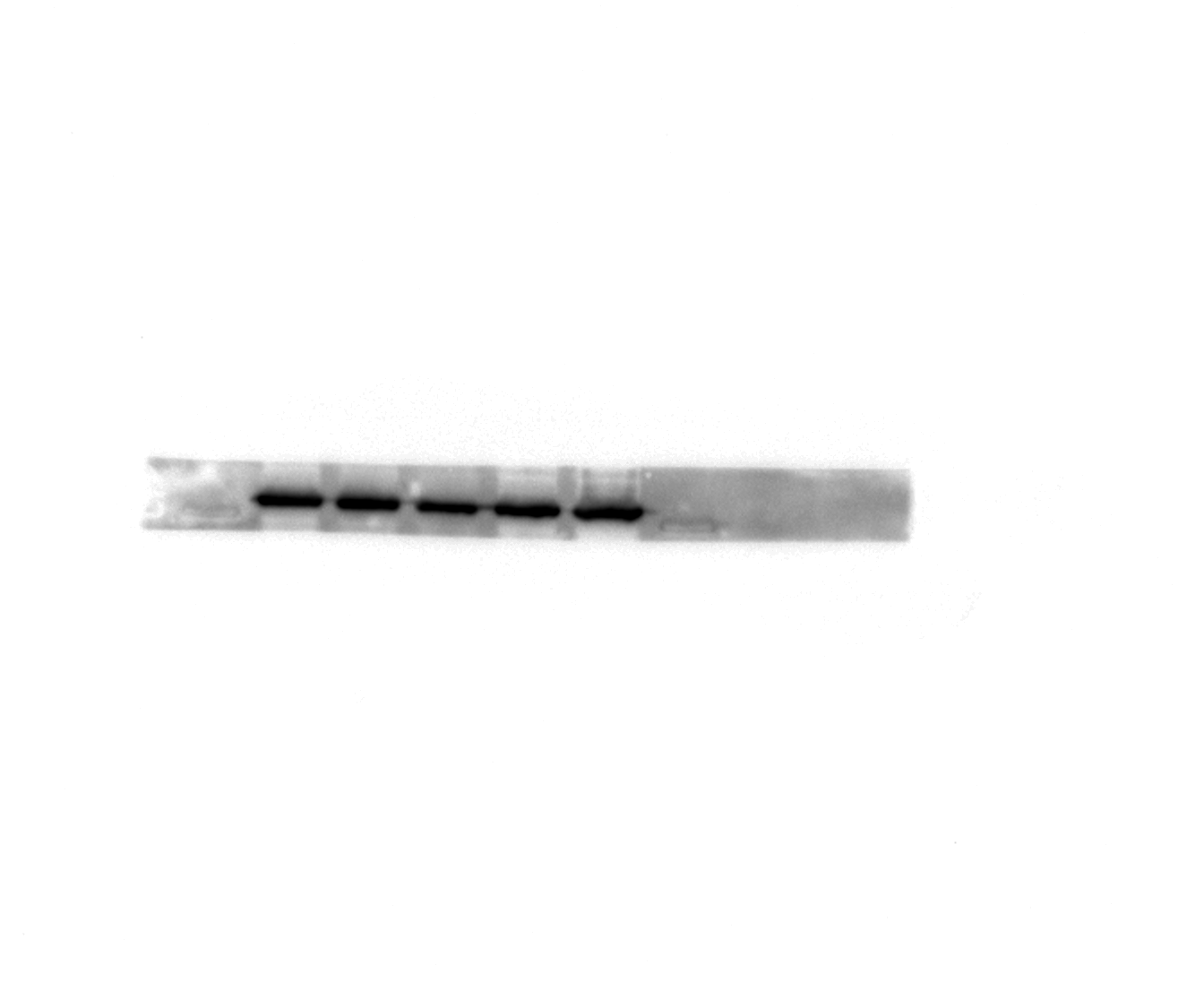

Supplement: Supplementary file 5 [file DataSheet1.zip › WB/敲低过表达/gap-2.Tif]

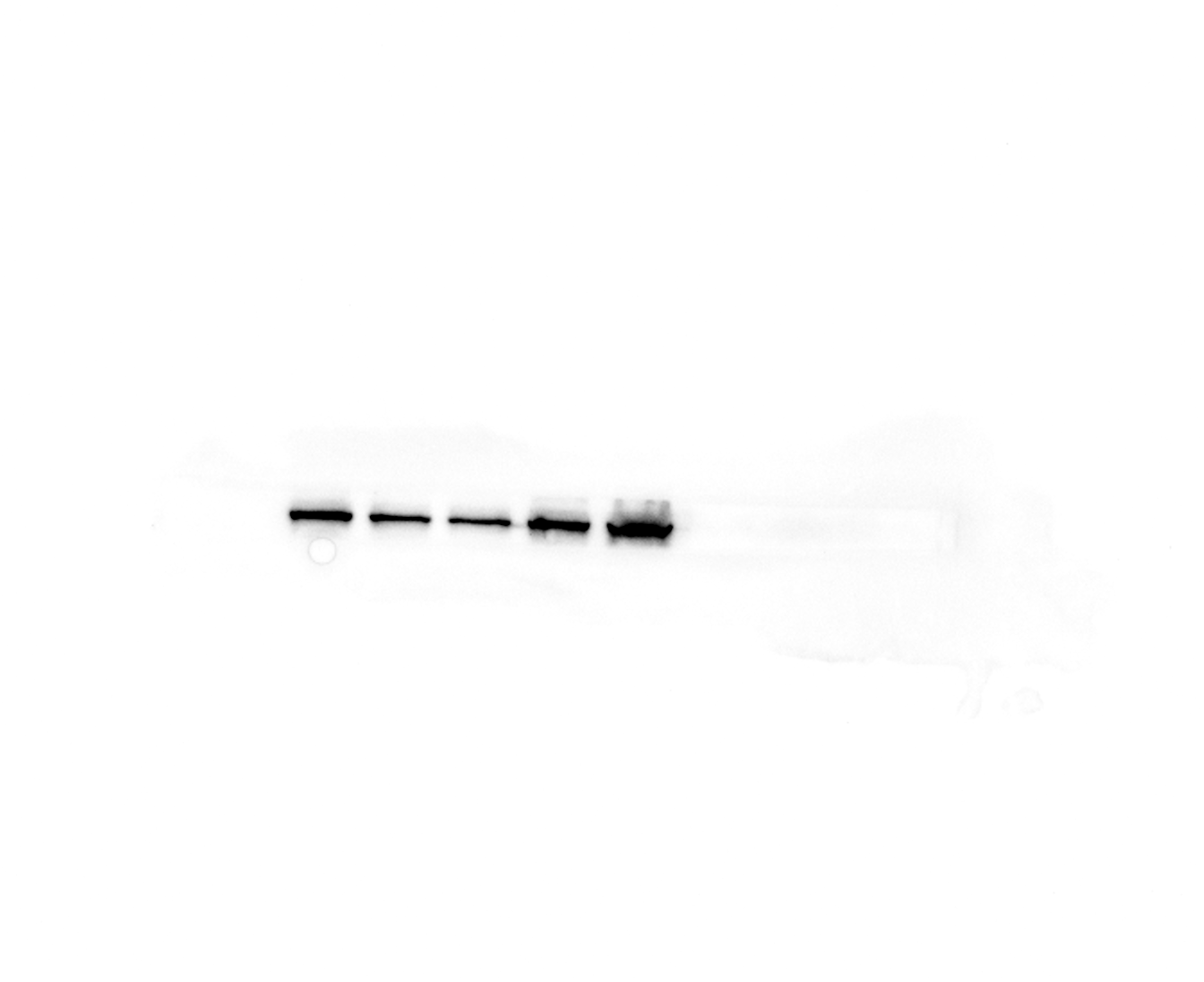

Supplement: Supplementary file 5 [file DataSheet1.zip › WB/敲低过表达/notch3 (2).Tif]

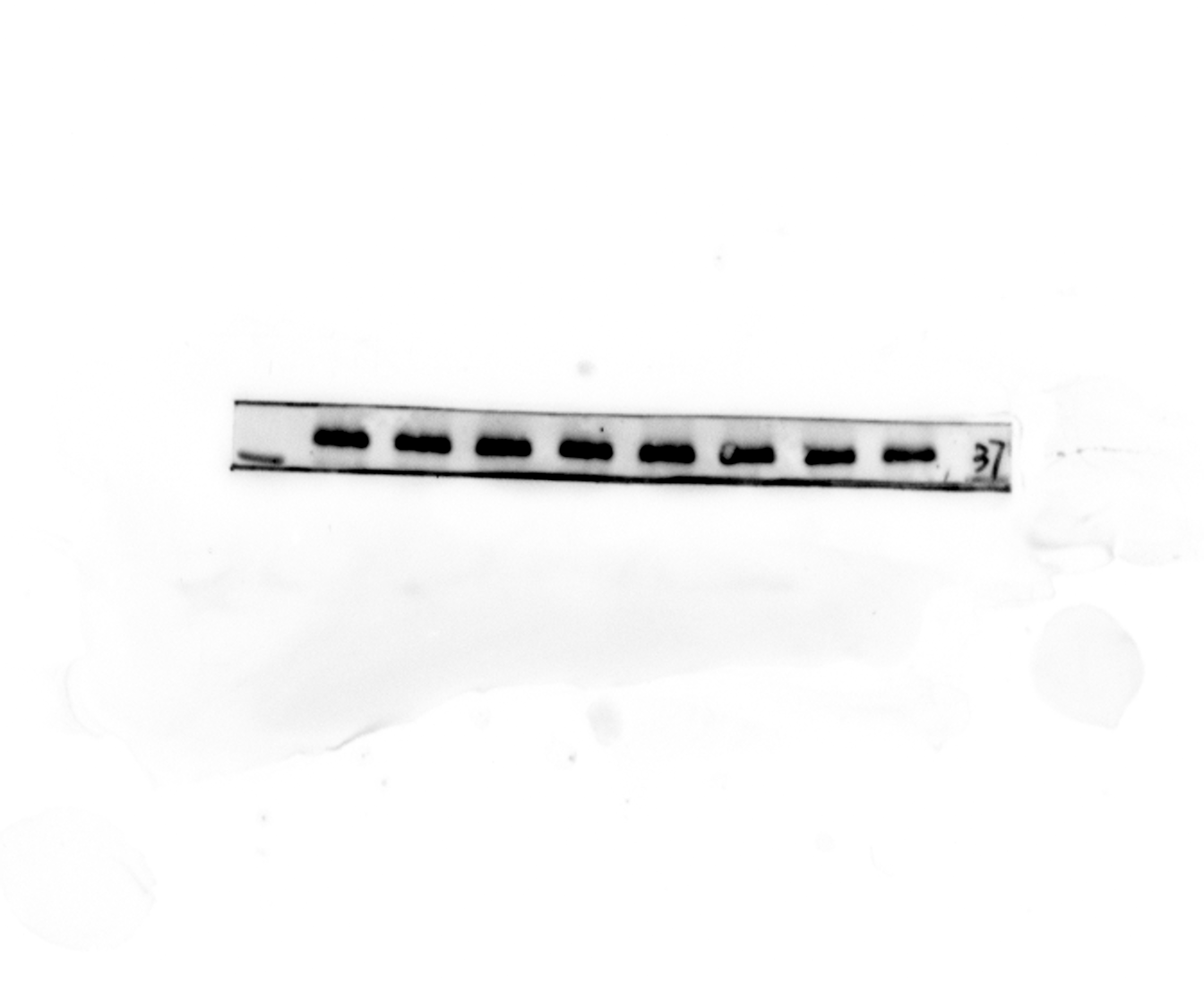

Supplement: Supplementary file 5 [file DataSheet1.zip › WB/组织/37-3.Tif]

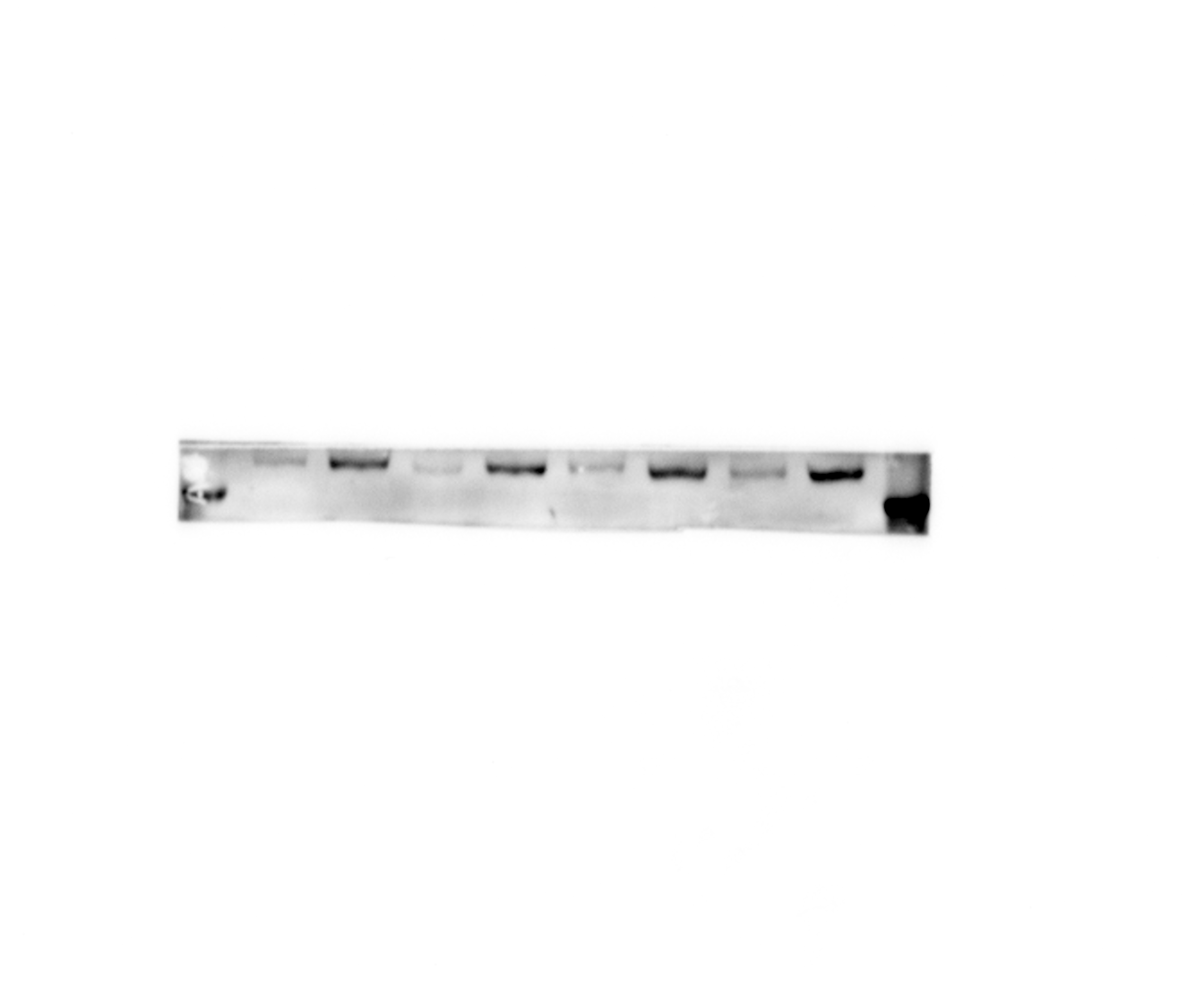

Supplement: Supplementary file 5 [file DataSheet1.zip › WB/组织/NOTCH3.Tif]

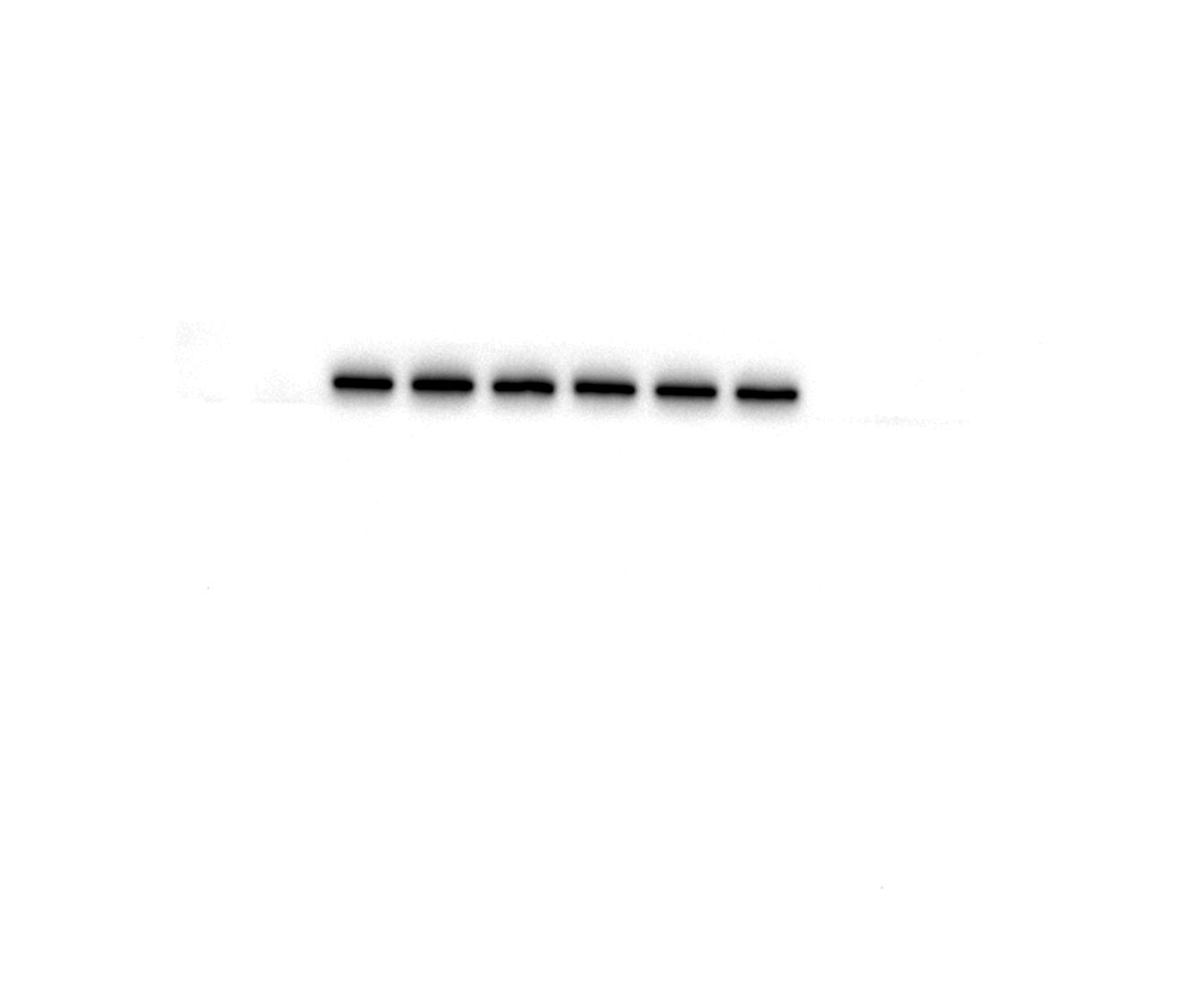

Supplement: Supplementary file 5 [file DataSheet1.zip › WB/细胞系WB/GAP.Tif]

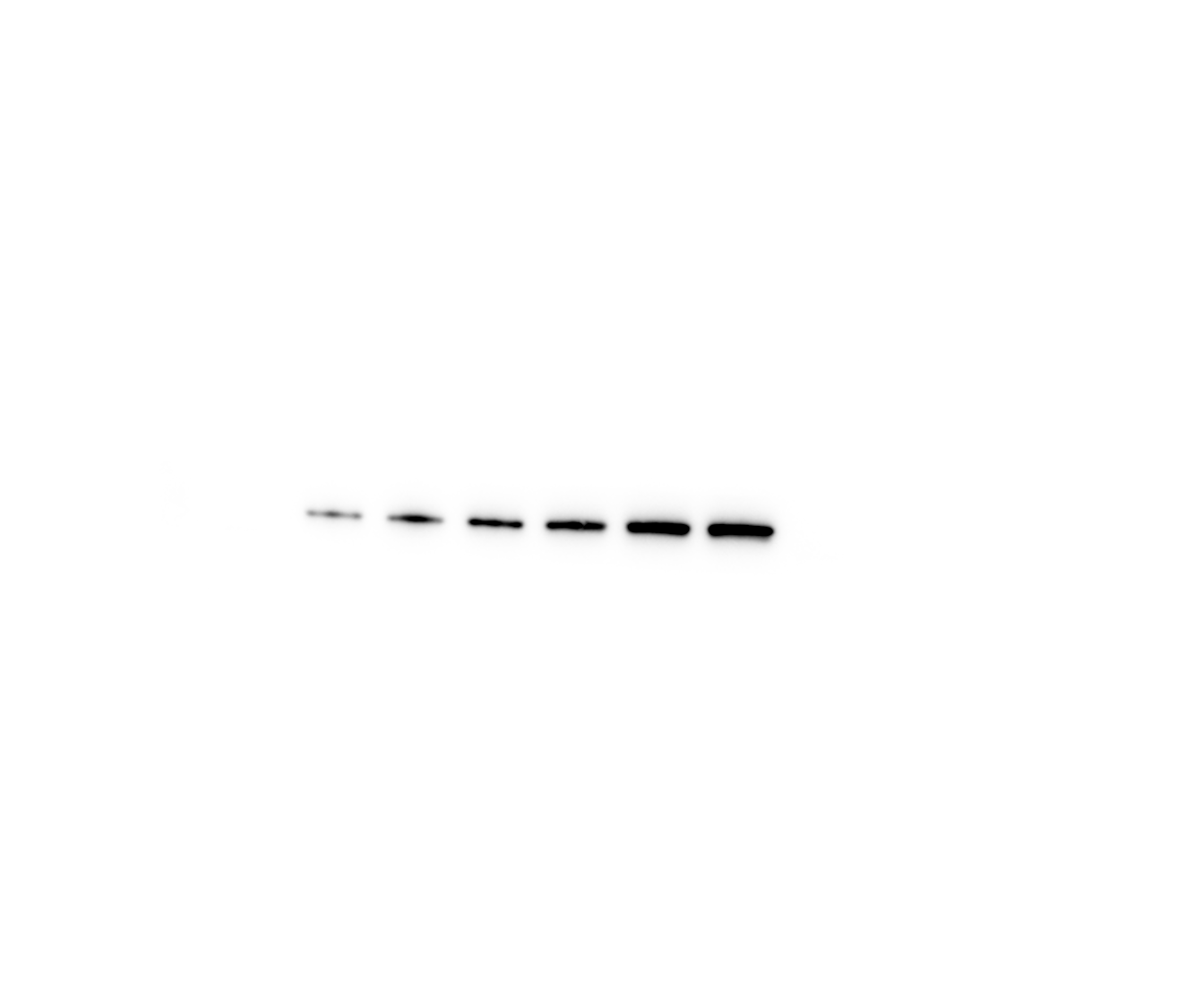

Supplement: Supplementary file 5 [file DataSheet1.zip › WB/细胞系WB/NOTCH3.Tif]

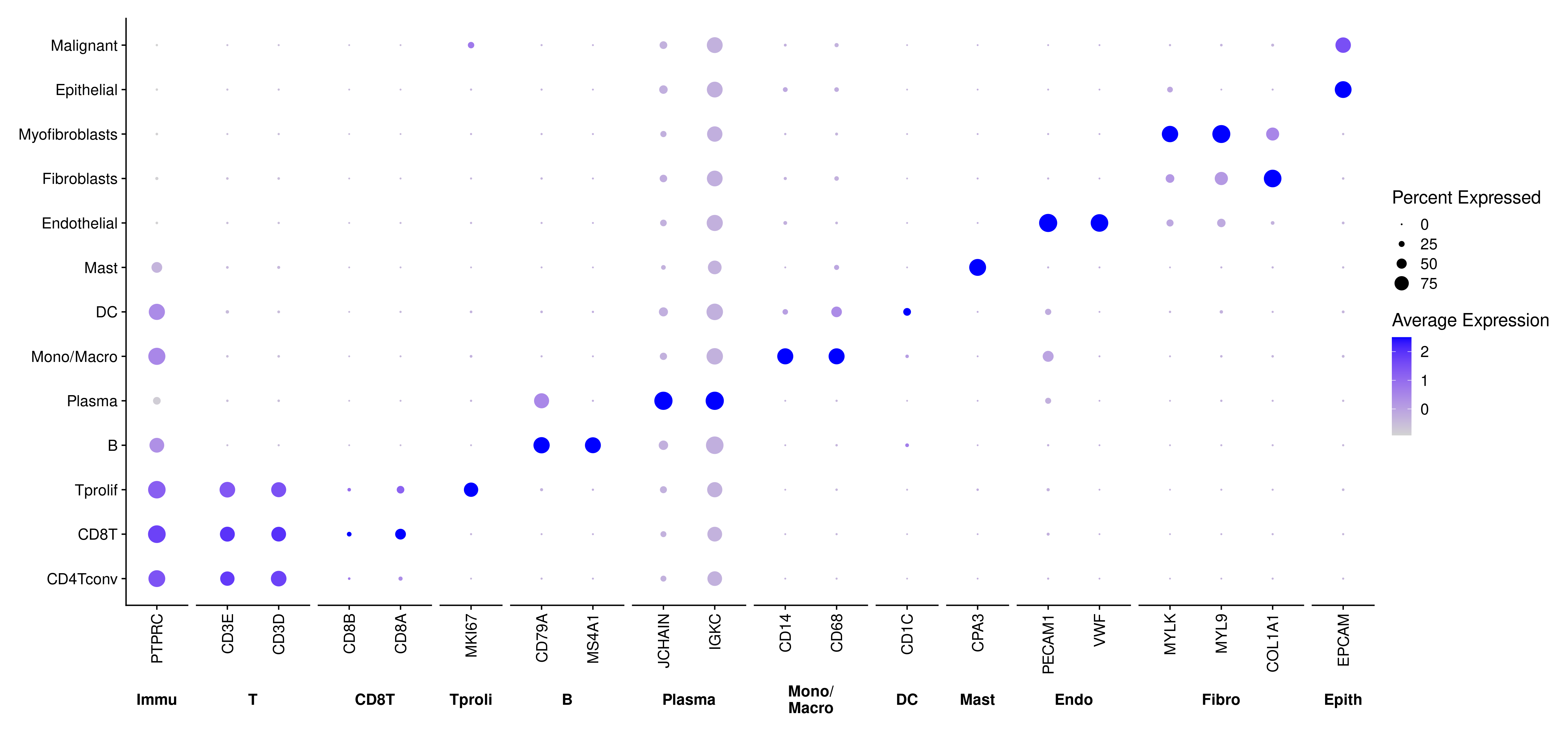

Supplement: Supplementary file 7 [file DataSheet3.zip › Figure 1/CRC_GSE166555_Dotplot.png]

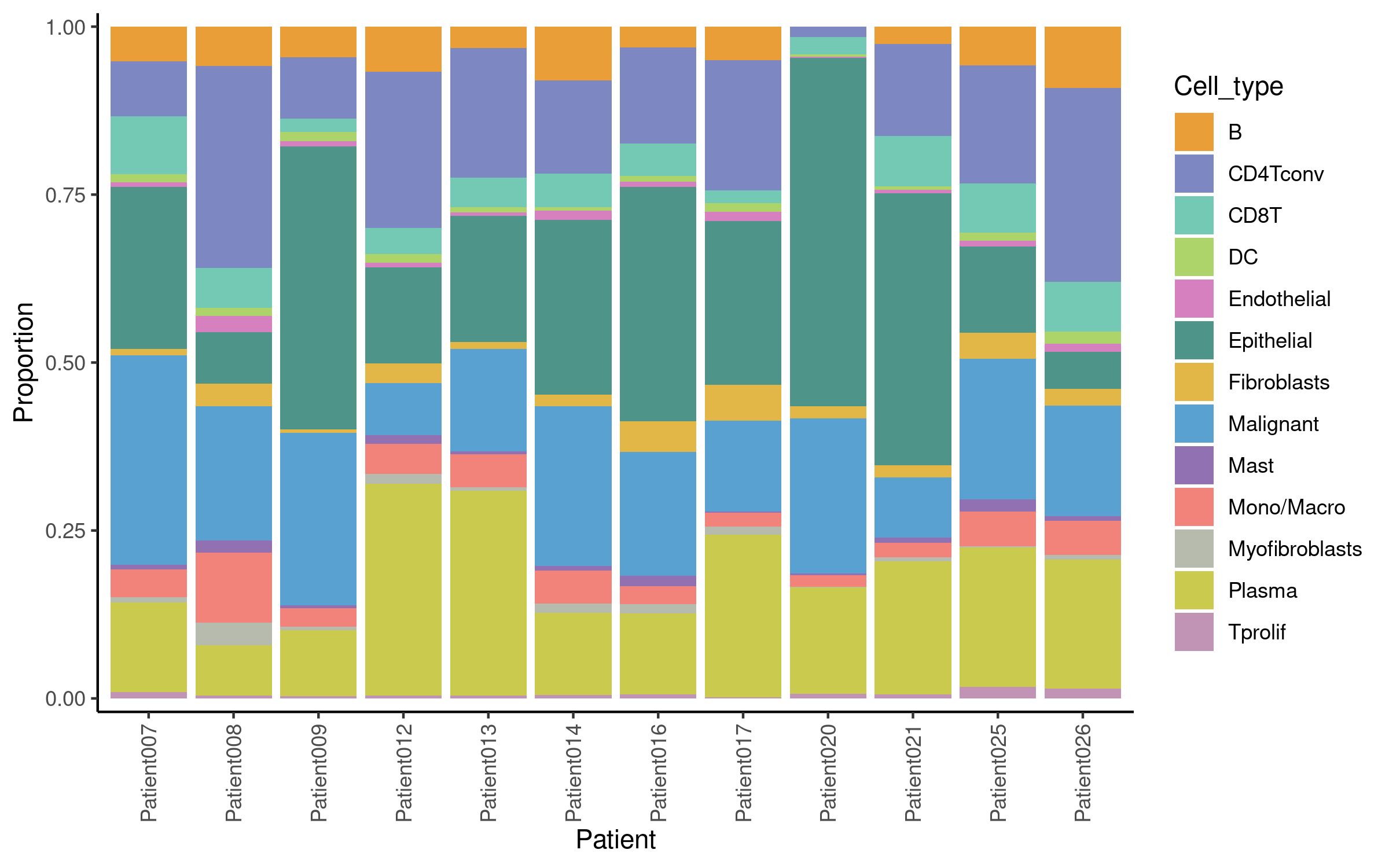

Supplement: Supplementary file 7 [file DataSheet3.zip › Figure 1/CRC_GSE166555_barplot.png]

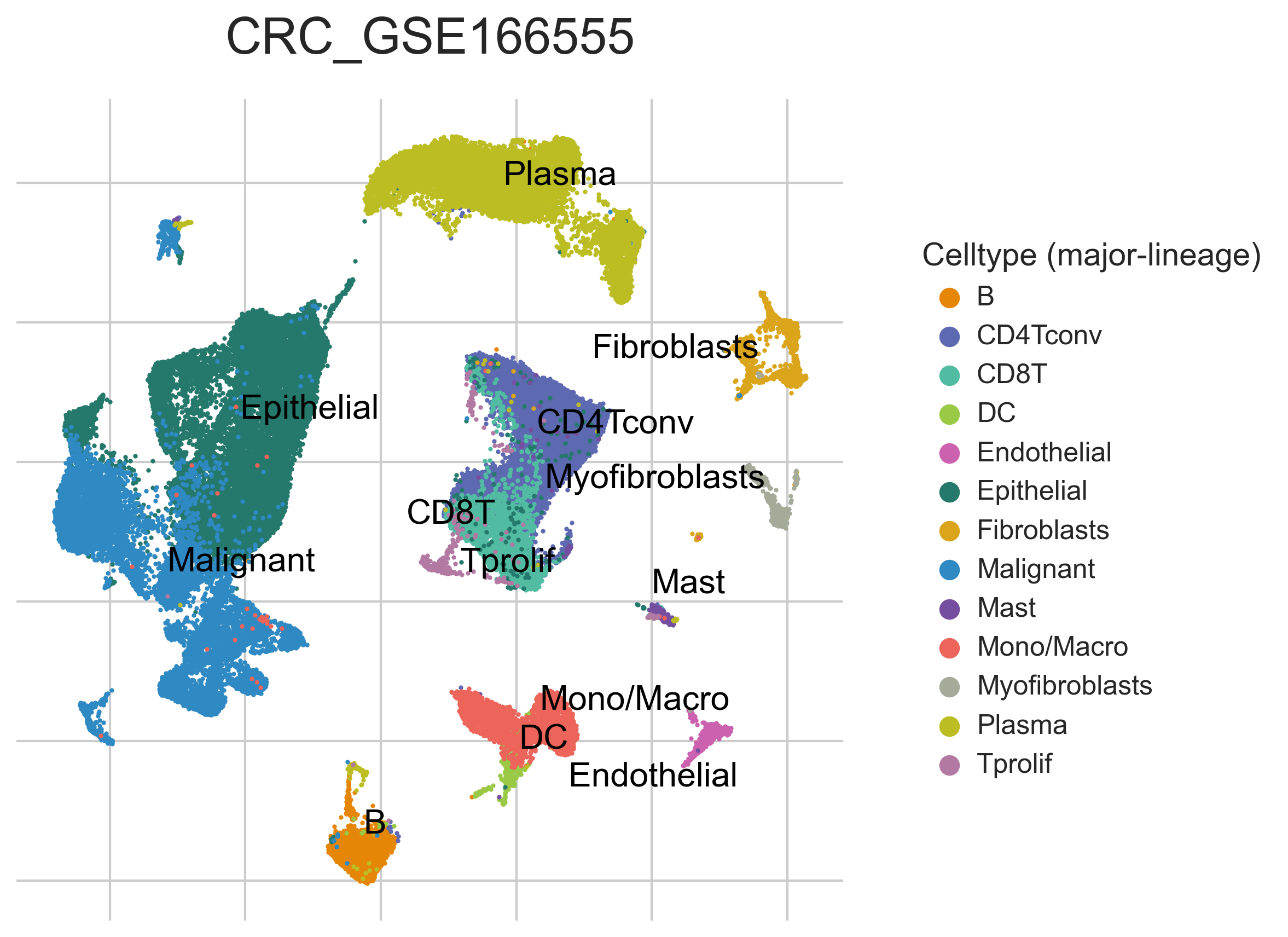

Supplement: Supplementary file 7 [file DataSheet3.zip › Figure 1/CRC_GSE166555_umap_Celltype_curated.png]
