# Supplementary material for: Exploiting branched-chain amino acid metabolism and NOTCH3 expression to predict and target colorectal cancer progression
Source: Front Immunol. 2024 Sep 2;15:1430352. doi: 10.3389/fimmu.2024.1430352 (PMC11402679; doi:10.3389/fimmu.2024.1430352)

	pvalue	Hazard ratio
NOTCH3	0.027	1.025(1.003−1.048)
HMGCL	0.031	0.933(0.876−0.994)
NOTCH4	0.034	1.189(1.013−1.395)
IVD	0.044	1.039(1.001−1.079)
NOTCH1	0.124	1.017(0.995−1.038)
SLC7A5	0.134	1.005(0.998−1.012)
HADHA	0.469	1.005(0.992−1.018)
ACADS	0.479	0.994(0.977−1.011)
HSD17B10	0.584	0.999(0.995−1.003)
MYC	0.643	0.998(0.992−1.005)
ALDH6A1	0.698	0.981(0.888−1.082)
ACAT1	0.779	0.992(0.938−1.050)
SLC1A5	0.956	1.000(0.997−1.003)
SLC7A8	0.963	1.000(0.981−1.020)

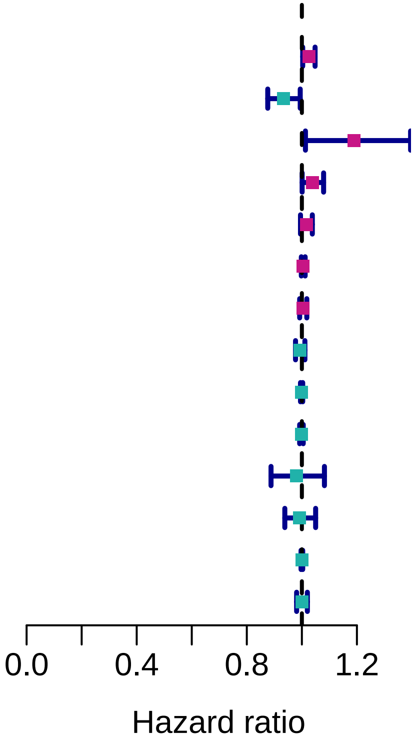

Supplement: Supplementary file 6 [file DataSheet2.zip › Figure 2-Raw data/A/uniCox.pdf]
