# Supplementary material for: Exploiting branched-chain amino acid metabolism and NOTCH3 expression to predict and target colorectal cancer progression
Source: Front Immunol. 2024 Sep 2;15:1430352. doi: 10.3389/fimmu.2024.1430352 (PMC11402679; doi:10.3389/fimmu.2024.1430352)
Supplement: Supplementary file 12 [file Table1.docx]

***Cell culture and transfection***

Five COAD cell lines (HT-29, Caco-2, Lovo, HCT 116, and DLD-1) and normal intestinal epithelial cells (NCM460) were obtained from the Cell Bank of the Chinese Academy of Sciences (Shanghai, China). All cell lines were maintained in DMEM medium (Gibco, USA). 10% fetal calf serum (Gibco, USA), 1% penicillin and streptomycin (Gibco, USA) were added to the medium. All were cultured at 37°C in a humidified atmosphere containing 5% CO_2_.

To establish stable NOTCH3 knockdown and NOTCH3 overexpression COAD cells, we purchased lentiviral vectors containing short hairpin RNA (shRNA) sequences targeting NOTCH3 (shNOTCH3-1 and shNOTCH3-2) from GenePharma (GenePharma, Shanghai, China) and NOTCH3 overexpression lentiviral vector (NOTCH3-OV) and their control vectors (shNC and Vector). sh-NC: UUCUCCGAACGUGUCACGUTT; sh-NOTCH3-1: CTGTGACACAAATCCGGTGAA; sh-NOTCH3-2: GCAUCUGCCAUGGAGGAUATT.

***Quantitative real-time PCR (qRT-PCR)***

A previous study described specific methods for total RNA extraction and quantitative real-time PCR (qRT-PCR). The following primers were used in this study: NOTCH3 forward, 5'-GTGTGTGTCAATGGCTGGAC-3' and NOTCH3 reverse, 5'-GTGACACAGGAGGCCAGTCT-3'; GAPDH forward, 5'-ACCTGCCAAATATGATGAC-3' and GAPDH reverse, 5'-ACCTGGTGCTCAGTGTAG-3’. GAPDH was used as an internal control. The procedures were performed three times to ensure accuracy and precision. The relative expression levels were calculated using the 2^−ΔΔCT^ method.

***Western blot assay***

Methods for protein extraction and Western blotting were described in detail in a previous study. NOTCH3 antibody and GAPDH antibody were purchased from Cell Signaling Technology (CST) with product numbers 2889 and 5174 respectively.

***Colony formation assay***

1000 cells were seeded into each well of a six-well plate and cultured in a humidified incubator with 5% CO2 for 2 weeks. Cells were then washed with PBS (phosphate-buffered saline), fixed with 4% paraformaldehyde for 10 min, and stained with 0.5% crystal violet solution for an additional 20 min. Afterwards, the colonies were counted and examined. The assay was performed three times.

***5-Ethynyl-2’-deoxyuridine (EdU) assay***

The cells were evenly plated on a 96-well plate at a concentration of 10,000 cells/100 μl culture medium per well. After 24 hours, aspirate the culture medium in the wells, add 50 μM EdU reaction solution, and incubate at 37°C for 2 hours. Subsequently, it was fixed with 4% paraformaldehyde for 30 minutes, the membrane was broken with 0.5% Triton x-100, and 1×Apollo® reaction cockail (100μL) was added for 30 minutes. The nuclei were then stained with 1×Hoechst 33342 for half an hour. Observe and take pictures under a fluorescence microscope.

***Wound healing assay***

In the wound healing assay, when the cell density in the well plate reaches about 90%, a linear wound is created at the bottom of the dish using a 200μL sterile pipette tip. After washing twice with PBS, the wound is observed under an inverted microscope and photographs are taken at fixed points. Then, 2ml of serum-free medium is added to culture the cells to exclude the effects of cell proliferation. After 24 hours, the cell growth status is observed, and at 48 hours the medium is aspirated and discarded. 1ml of PBS is added to prevent the cells from dehydrating and shrinking, and photographs are taken at the same location again. Subsequently, the area between the two sides of the scratch is measured using ImageJ software, and the cell invasion ratio is calculated.

***Transwell assay***

In a Transwell assay, 500μL of medium containing 10% serum is added to a 24-well plate, and a transwell insert coated with matrix gel is placed in the well. Then, 20,000 cells in 200μL of serum-free medium are added directly into the center of the insert. After 24-36 hours, the insert is removed, and washed twice with PBS. The cells on the upper layer of the insert are then scraped off using a cotton swab. Subsequently, the insert is soaked in crystal violet staining solution for half an hour. After photographing under a microscope, the cells are counted.

***Animal assay***

We purchased 4-week-old female nude mice from the Experimental Animal Center of Nanjing Medical University and raised them under SPF conditions for animal experiments. Animal experimental operations comply with experimental animal welfare ethics. The specific operation is to subcutaneously inject COAD cells that stably knock down or overexpress NOTCH3 (1×106 cells/100μL PBS) into the middle and upper parts of the groins on both sides of nude mice. The subcutaneous tumor volume was observed and measured once a week, and we used the formula (length*width)/2 to estimate the mouse tumor volume. After 4 weeks, the nude mice were sacrificed, the tumor volume and weight were measured, and the tissues were stained with Ki67. For the lung metastasis model, we injected COAD cells (0.5 × 10 cells/100μL PBS) into the tail vein of nude mice. After 4 weeks, the nude mice were sacrificed and dissected to observe the lung metastasis. Fresh lungs were taken out for photography, and then the lungs tissues were stained with Hematoxylin and eosin (HE).

***Cell function recovery assays***

For cell function recovery assays, branched chain alpha-ketoacids (BCKAs ) including 4-methyl 2-oxopentanoic acid sodium salt (KIC, W387101, Sigma-Aldrich), sodium-3-methyl-2-oxobutyrate (KIV, 198994, Sigma-Aldrich), 3-methyl-2-oxovaleric acid sodium salt (KMV, 198978, Sigma-Aldrich) were added at a concentration of 0.5mM.
